# Competitive Anion Anchoring and Hydrogen Bonding in Multiscale‐Coupling Composite Quasi‐Solid Electrolytes for Fire‐Safety and Long‐Life Lithium Metal Batteries

**DOI:** 10.1002/advs.202501012

**Published:** 2025-03-24

**Authors:** Ding Hu, Guo‐Rui Zhu, Ping‐Hui Duan, Si‐Chong Chen, Gang Wu, Yu‐Zhong Wang

**Affiliations:** ^1^ The Collaborative Innovation Center for Eco‐Friendly and Fire‐Safety Polymeric Materials (MoE) National Engineering Laboratory of Eco‐Friendly Polymeric Materials (Sichuan) State Key Laboratory of Polymer Materials Engineering College of Chemistry Sichuan University Chengdu Sichuan 610064 China

**Keywords:** anions anchoring, composite quasi‐solid electrolytes, fire safety, hydrogen bonding, lithium‐metal batteries

## Abstract

Composite solid‐state electrolytes (CSEs) using Li_1+x_Al_x_Ti_2‐x_(PO_4_)_3_ (LATP) as active fillers offer promising prospects for large‐scale lithium metal batteries (LMBs) applications due to their high environmental stability, cost‐effectiveness, and improved safety. However, the challenges persist owing to high interfacial resistance with electrodes and instability with lithium metal. Herein, self‐assembly nanofiber/polymers/LATP composite quasi‐solid electrolytes (SL‐CQSEs) are reported through in situ polymerization of precursor solution containing vinylene carbonate (VC), fluoroethylene carbonate (FEC), lithium bis(trifluoromethanesulfonic) imide (LiTFSI) in a porous and flexible self‐supporting skeleton (SSK) consisting of 2‐(3‐(6‐methyl‐4‐oxo‐1,4‐dihydropyrimidin‐2‐yl)ureido)ethyl methacrylate (UPyMA)’s self‐assembly nanofiber (SAF), poly(vinylidene fluoride‐co‐hexafluoropropylene) (PVDF‐HFP) and LATP. Anion‐anchoring/hydrogen‐bonding competition and intercomponent multiscale‐coupling effects on SL‐CQSEs are found, which contribute to their incombustibility, excellent room‐temperature ionic conductivity (1.03 mS cm^−1^), wide electrochemical window (5.1 V), good interfacial compatibility, and lasting inhibition of lithium dendrites. LiFePO_4_/Li cells with SL‐CQSEs not only exhibit high‐rate performance and long‐term cycling stability, with a capacity retention of 90.4% at 1C and 87% even at 4C after 1000 cycles, but also can resist fire and mechanical abuse, highlighting the potential applications of SL‐CQSEs for high‐performance and safety LMBs.

## Graphical abstract

A novel self‐assembly fiber (SAF)/polymers/LATP composite quasi‐solid electrolytes based on TFSI^‐^ anchoring and intermolecular hydrogen bonding competitive and multiscale coupling effects, named SL‐CQSE, which possesses the perfect reconciliation of facilitating Li^+^ migration, inducing stable SEI layer construction, and strengthening mechanical properties, is in situ prepared. Non‐inflammable CQSE‐20 and anions‐induced Li_3_N/LiF‐rich inorganic/organic hybrid SEI endow lithium batteries with excellent electrochemical performance and superior fire safety.

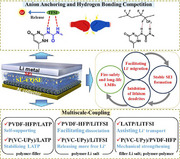

## 1 Introduction

Over the past 20 years, lithium batteries (LBs) have been widely found in many technological devices from smartphones to electric vehicles (EVs).^[^ ^1^ ^]^ Despite extensive real‐world applications, LBs are still limited by many severe issues, especially the frequent safety incidents such as leakage, spontaneous combustion, and even explosion of the flammable and volatile liquid electrolyte in them.^[^ ^2^ ^]^ Solid‐state electrolytes (SSEs) are expected to solve the safety issues of traditional LBs because they are not prone to setting on fire as easily as liquid electrolytes do.^[^ ^3^ ^]^ In addition, SSEs might also alleviate another concern about irrepressible growth of lithium dendrites in liquid electrolytes. This helps endow LBs with higher energy densities by matching the SSEs with the lithium metal anode to replace graphite,^[^ ^4^ ^]^ that is lithium metal batteries (LMBs). SSEs can be split into two major classes: solid polymer electrolytes (SPEs) and ceramic electrolytes (CEs). Compared to CEs, SPEs offer the advantages of excellent processability, high flexibility, and good contact with the electrodes. However, the main obstacle of lower ionic conductivity hinders their application at room temperature. One promising compromise is to introduce the inorganic fillers into SPEs to construct composite solid‐state electrolytes (CSEs).

Broadly, inorganic fillers come in two varieties. Inert fillers, such as aluminum oxide (Al_2_O_3_),^[^ ^5^ ^]^ silicon dioxide (SiO_2_),^[^ ^6^ ^]^ graphitic carbon nitride (g‐C_3_N_4_),^[^ ^7^ ^]^ metal–organic framework (MOF),^[^ ^8^ ^]^ and covalent organic framework (COF),^[^ ^9^ ^]^ would reduce the crystallinity of the polymer matrix, but themselves do not participate in the Li‐ion transport. Active fillers, such as Li_3x_La_2/3‐x_TiO_3_ (LLTO),^[^ ^10^ ^]^ Li_7_La_3_Ta_2_O_12_ (LLZO),^[^ ^11^ ^]^ and Li_1+x_Al_x_Ti_2‐x_(PO_4_)_3_ (LATP),^[^ ^12^ ^]^ are also often used for ceramic electrolytes. Among them, LATP has been widely studied owing to its low cost, high air and water resistance,  high ionic conductivity at room temperature (>10^−4^ S cm^−1^), and wide electrochemical window (>5 V).^[^ ^13^ ^]^ Despite the above advantages, the chemical incompatibility of LATP and lithium metal is a pressing question, where spontaneous reduction of Ti^4+^ induces the generation of reactive interphases with an electronic conductivity, so‐called mixed conducting interphases (MCI),^[^ ^14^ ^]^ which further results in uncontrolled side reactions to deteriorate electrode/electrolyte interfaces. To address this issue, various protective layers were adopted to prevent direct contact between LATP and lithium metal, including metal,^[^ ^15^ ^]^ metal oxide,^[^ ^16^ ^]^ polymer,^[^ ^17^ ^]^ etc. Although the protective layer has effectively avoided physical contact between LATP with lithium metal, the preparation methods are complicated, time‐consuming, and commercially inapplicable,^[^ ^18^ ^]^ such as atomic layer deposition (ALD),^[^ ^19^ ^]^ and magnetron sputtering.^[^ ^20^ ^]^ Therefore, it is crucial to stabilize the interface using a facile, simple, and low‐cost strategy.

On the other hand, the deep interfacial resistance from the poor solid‐solid interfacial contact between electrolyte and electrodes leads to insufficient ion transport. Accordingly, the ionic conductivity of solid‐state electrolytes is generally lower than 10^−3^ S cm^−1^, which hardly competes with that of the practical liquid electrolytes.^[^ ^21^ ^]^ Recently, in situ polymerization composite quasi‐solid electrolytes (CQSEs) have demonstrated huge potential in optimizing the interfacial contact and ionic conductivity of CSEs. CQSEs and in situ polymerization technique can take into account the advantages of solid‐liquid, partially solving the interface problem in the cycle, and enabling battery systems to combine high performance with manufacturing flexibility.^[^ ^22^ ^]^ For example, Zha et al. prepared an ice‐templated Li_1.5_Al_0.5_Ge_1.5_(PO_4_)_3_ (LAGP) scaffold and then fabricated the vertically aligned ceramic/polymer hybrid electrolyte via in situ polymerization.^[^ ^26^ ^]^ Jin et al. prepared silane modified‐Li_1.3_Al_0.3_Ti_1.7_(PO_4_)_3_ (Si@LATP)/poly(vinylidene fluoride) (PVDF) composite nanofiber membranes by electrospinning method, then constructed quasi‐solid electrolytes through in situ polymerization.^[^ ^27^ ^]^ Although some progress has been made, it is highly desired and still challenging to achieve further breakthroughs in the performance of CQSEs‐based LMBs through the multiscale coupling effects on polymer‐filler, polymer‐lithium salt, and filler‐lithium salt.

**Figure 1:**
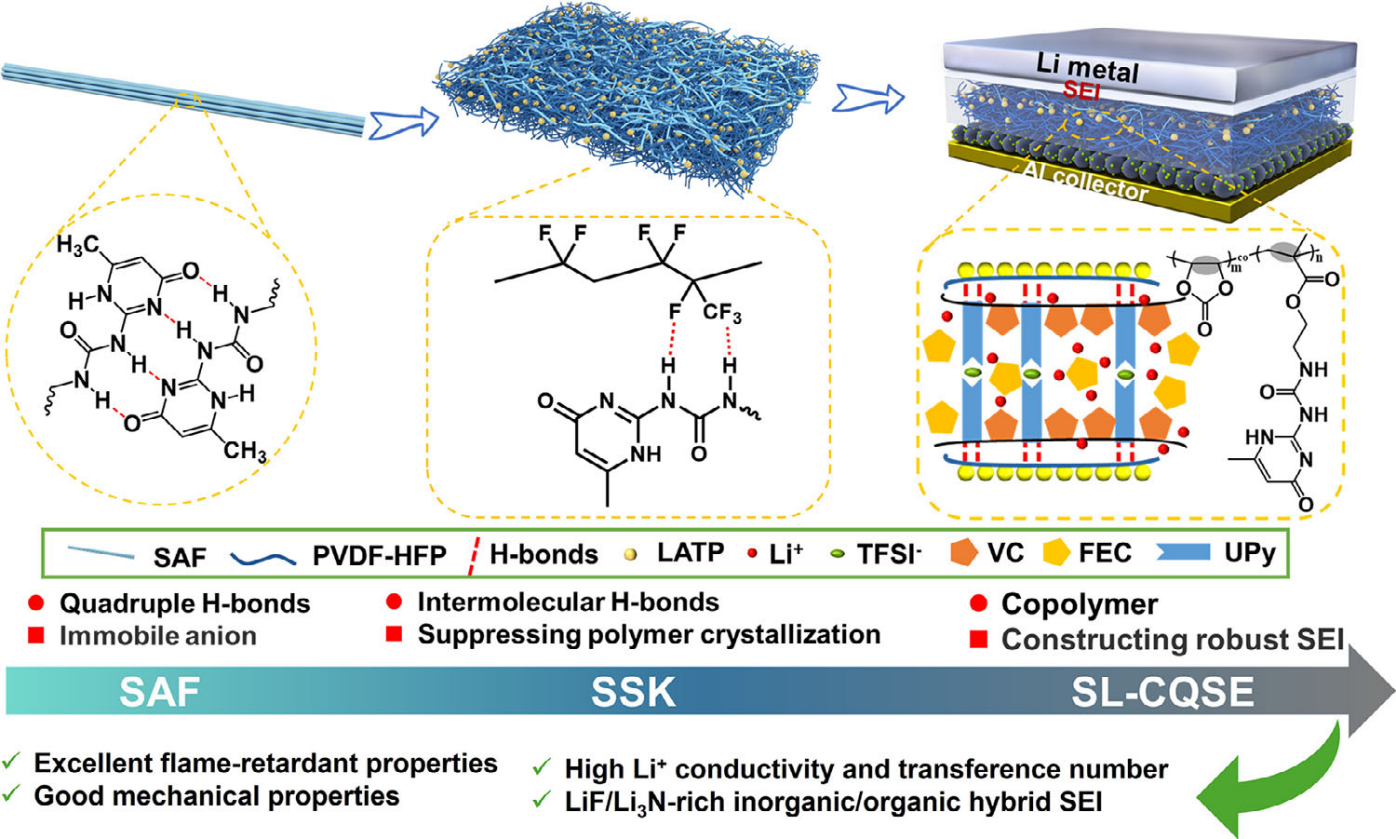
Schematic illustration of the SL‐CQSEs structures, internal molecular interactions, and their advantages.

In this work, we proposed a simple and efficient method to prepare a porous and flexible 3D self‐supporting skeleton (SSK) consisting of 2‐(3‐(6‐methyl‐4‐oxo‐1,4‐dihydropyrimidin‐2‐yl)ureido)ethyl methacrylate (UPyMA) self‐assembly fiber (SAF), poly(vinylidene fluoride‐co‐hexafluoropropylene) (PVDF‐HFP) and LATP. Upon in situ polymerization, UPyMA in SSK was copolymerized with vinylene carbonate (VC) to form multiscale coupling SAF/polymers/LATP composite quasi‐solid electrolytes, named as SL‐CQSEs. Thus, the intermolecular hydrogen bonds between SAF and PVDF‐HFP were weakened by the anchoring interaction between UPy units and TFSI^−^ anions, and competition between anions anchoring and intermolecular hydrogen bonds was established (**Figure** **1**). Competitive and coupling effects can realize the perfect reconciliation of facilitating lithium‐ion (Li^+^) migration, inducing stable SEI layer construction, and strengthening mechanical properties. As a result, the SL‐CQSEs CQSE‐20 exhibits a high ionic conductivity of 1.06 mS cm^−1^ at 25 °C, a Li^+^ transference number (t_Li+_) of 0.63, and a wide electrochemical window of up to 5.1 V. The steady cycle life of the Li/Li symmetric cell is extended to 750 h without dendrite formation. Because anions‐induced Li_3_N/LiF‐rich inorganic/organic hybrid SEI structure stabilizes the interface layer and makes uniform deposition of Li, the LFP/Li cells deliver excellent high‐rate performance and long‐term cycling stability, with the initial discharge capacity of 147.4 mAh·g^−1^ and capacity retention of 90.4% over 1000 cycles at 1C, and even at 4C, the initial discharge capacity of 121.8 mAh·g^−1^ and capacity retention of 87% over 1000 cycles. In addition, the SL‐CQSEs are completely non‐inflammable, indicating that it is very promising to further endow the high‐performance LMBs with superior fire safety.

## 2 Results and Discussion

### 2.1 Fabrication and Characterization of SL‐CQSEs

Figure S1 (Supporting Information) illustrates the preparation process of SL‐CQSEs, which involves two main steps, i.e., 1) fabrication of SSKs and 2) in situ polymerization of precursor solution in SSKs to obtain SL‐CQSEs. Firstly, UPyMA was mixed with PVDF‐HFP and LATP in N‐methyl‐2‐pyrrolidone to fabricate SSKs with a different mass content of UPyMA (i.e., 0 wt%, 15 wt%, 20 wt%, and 25 wt%), named SSK‐0, SSK‐15, SSK‐20, and SSK‐25, by blade coating and phase separation process in turn. PVDF‐HFP acts as both a binder and forms intermolecular hydrogen bonds with UPyMA within the SSKs. The digital photographs confirm that the SSK‐20 is a free‐standing and flexible white membrane (Figure S1a, Supporting Information). Secondly, a precursor solution containing vinylene carbonate (VC), fluoroethylene carbonate (FEC), lithium bis(trifluoromethanesulfonic)imide (LiTFSI), and 2,2′‐azobis(2‐methylpropionitrile) (AIBN) was injected into the SSKs, and then the copolymerization between UPyMA and VC was initiated thermally to obtain relevant SL‐CQSEs, named as CQSE‐0, CQSE‐15, CQSE‐20, CQSE‐25, respectively (Figures S1b, Supporting Information).

**Figure 2:**
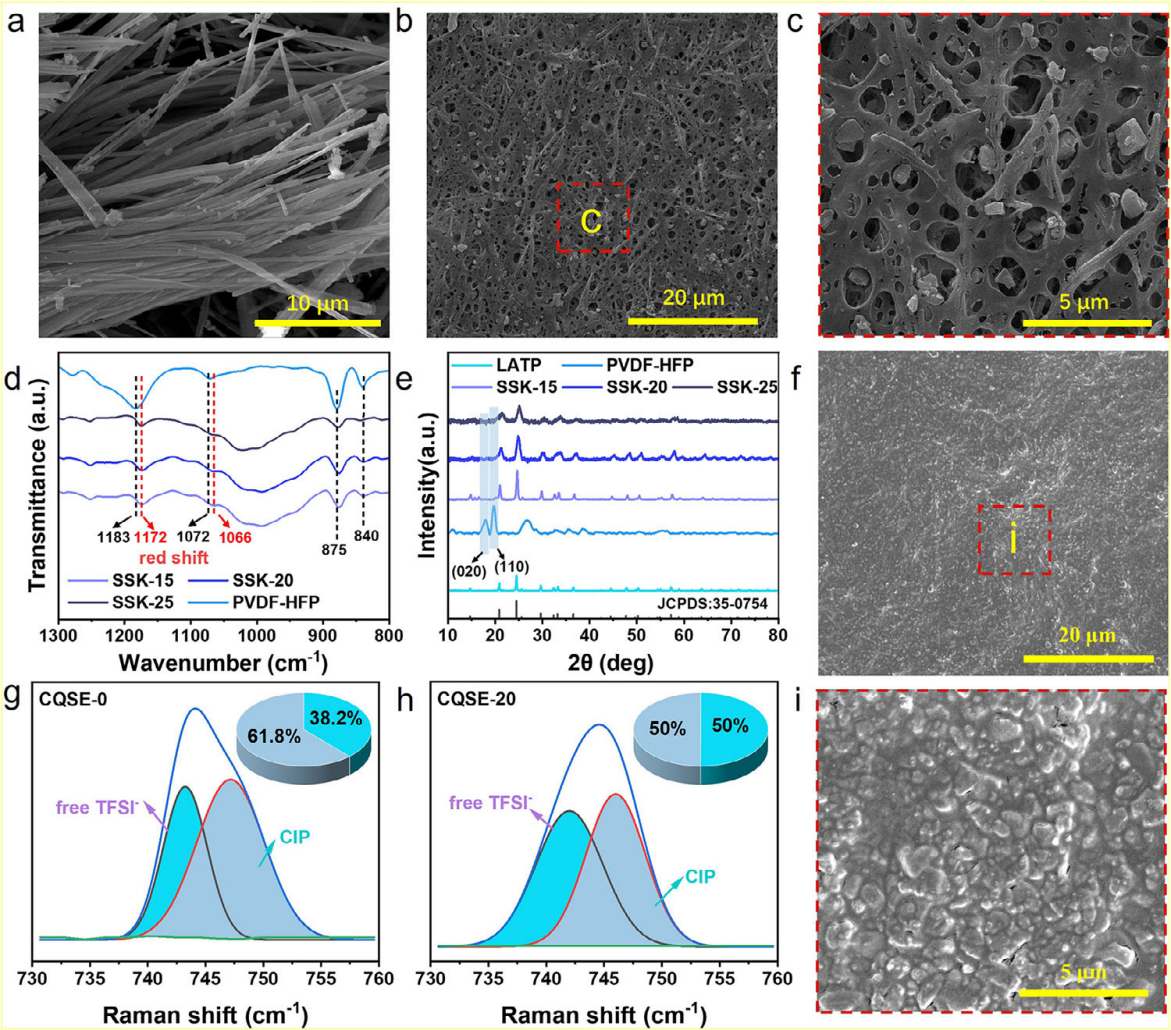
a) SEM images of UPyMA. b,c) Top‐view SEM images of SSK‐20. d) FT‐IR spectra of SSKs and PVDF‐HFP. e) XRD patterns of LATP, PVDF‐HFP, and SSKs. f,i) Top‐view SEM images of CQSE‐20. Raman spectra of g) CQSE‐0 and h) CQSE‐20.

The successful synthesis of UPyMA (Figure S2, Supporting Information) is confirmed by ^1^H and ^13^C nuclear magnetic resonance (NMR) spectra (Figure S3, Supporting Information). Interestingly, scanning electron microscopy (SEM) image reveals that UPyMA can self‐assemble into nanofibers with a micrometer‐scale length (**Figure** **2**a). As a control, the pairwise mixtures of LATP, PVDF‐HFP, UPyMA three components, named as PVDF‐HFP/UPyMA, LATP/UPyMA, and PVDF‐HFP/LATP, were prepared and observed. It can be found from SEM images (Figure S4, Supporting Information) that PVDF‐HFP/UPyMA is a web‐like membrane containing nanofibers, there are short nanofibers and nanoparticles in LATP/UPyMA but cannot generate the self‐supporting membrane, and no nanofibers can be seen in PVDF‐HFP/LATP (named as SSK‐0). Top‐view SEM images show that pores, LATP nanoparticles, and short nanofibers are more well‐distributed in SSK‐20 compared to SSK‐15 and SSK‐25 (Figure 2b,c; Figure S5, Supporting Information). Cross‐sectional SEM images demonstrate that SSK‐20 has a thickness of 78 ± 5 µm, and energy‐dispersive X‐ray spectroscopy (EDS) mapping images further show even distribution of UPyMA, LATP, and PVDF‐HFP in SSK‐20 (Figure S6, Supporting Information). Mercury intrusion porosimetry (MIP) confirms the existence of micro‐nano pores structure and the increased porosity with increasing UPyMA content (Figure S7, Supporting Information). SEM images of SSK‐20 obtained by phase separation with different solvents show that using deionized water (DIW) as the solvent can endow the SSKs with the well‐maintained pores and fibrous structures (Figure S8, Supporting Information).

Furthermore, Fourier transform infrared (FT‐IR) spectroscopy was carried out to investigate hydrogen bond interactions in SSK. As shown in Figure 2d, the characteristic peaks at 875 and 840 cm^−1^ correspond to the bending vibration of CF_2_. The characteristic peaks at 1072 and 1183 cm^−1^ correspond to the symmetric stretching vibration of C‐F, which is red‐shifted by the addition of UPyMA, possibly implying the intermolecular hydrogen bonds between PVDF‐HFP and UPyMA. In addition, X‐ray diffraction (XRD) patterns of LATP, PVDF‐HFP, and SSKs indicate the presence of the rhombohedral NASICON‐type LiTi_2_(PO_4_)_3_ phase (JCPDS: 35–0754) (Figure 2e). The characteristic peaks of PVDF‐HFP at 18° and 20° reflect the presence of the crystalline α‐phase, while these two peaks are almost invisible for all SSKs, indicating that the UPyMA can inhibit the crystallization of PVDF‐HFP. The reason could be attributed to intermolecular hydrogen bonds between UPyMA and PVDF‐HFP.

Post‐polymerization, the SL‐CQSEs were first observed by SEM. CQSE‐20 surface is dense and smooth (Figure 2f,i), while CQSE‐0, CQSE‐15, and CQSE‐25 show inhomogeneity, with the exposed LATP nanoparticles or nanofibers (Figure S9, Supporting Information). Cross‐section SEM images of SL‐CQSEs in Figure S10 (Supporting Information) show that the CQSE‐20 contains suitable fibrous and porous structures compared with CQSE‐0, CQSE‐15, and CQSE‐25, which will be more favorable to provide multiple continuous Li^+^ transfer channels. The in situ polymerization was evaluated by electrochemical impedance spectroscopy (EIS) during the in situ polymerization process (Figure S11, Supporting Information). After heating at 60 °C for 10 h, the interface impedance of the LFP/Li battery tended to be constant, reflecting the completion of the in situ polymerization, so as to help the formation of a stable interface between CQSE‐20 and the electrode. Further evaluation of chemical structure changes of in situ polymerization was conducted by FT‐IR analyses (Figure S12, Supporting Information). The FT‐IR spectrum reveals characteristic vibration peaks for C═C bonds of VC (1565 cm^−1^), UPyMA (1654 cm^−1^), C═O bonds (1801 cm^−1^), and C─O─C bonds (1106 cm^−1^). In all SL‐CQSEs, the C═C bond peaks almost disappear, confirming nearly complete copolymerization between VC and UPyMA monomers. Optical photographs before and after polymerization visually demonstrate the polymerization process (Figure S13, Supporting Information). Subsequently, Raman analysis was used to characterize the coupling effect of SL‐CQSE on LiTFSI salt. The characteristic peaks at 743 and 748 cm^−1^ represent free TFSI^−^ and contact‐ion pair (CIP), respectively. Compared with CQSE‐0, the free TFSI^−^ content in CQSE‐20 is significantly higher, indicating that the resulted copolymer consisting of VC and UPyMA monomers, named as P(VC‐UPy), should have a certain anchoring effect with TFSI^−^, which helps a dissociation of lithium salt to release more free TFSI^−^.

**Figure 3:**
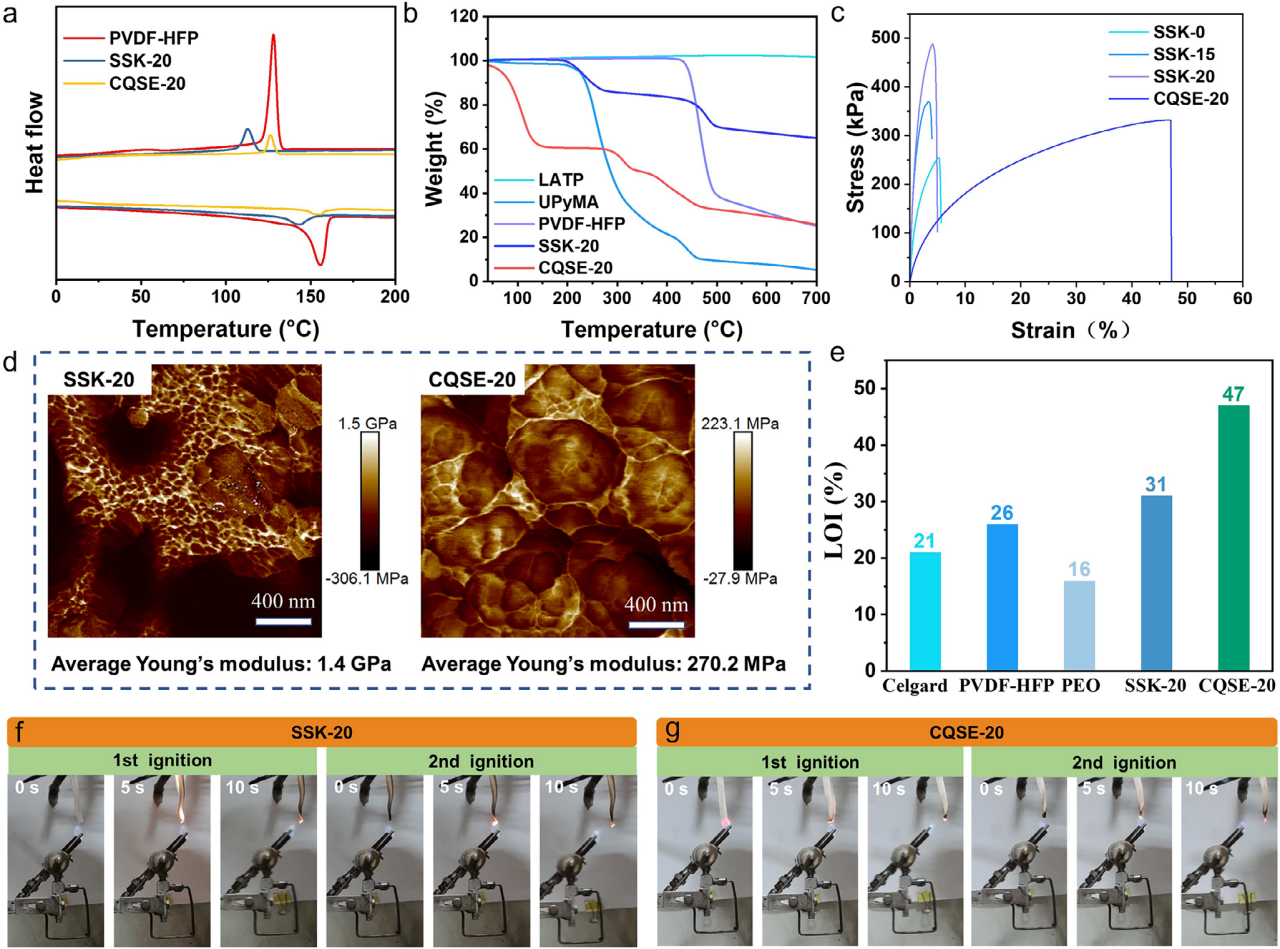
a) DSC curves of PVDF‐HFP, SSK‐20, and CQSE‐20. b) TGA curves of LATP, UPyMA, PVDF‐HFP, SSK‐20, and CQSE‐20. c) Stress−strain curves of various SSKs and CQSE‐20. d) AFM images of SSK‐20 and CQSE‐20. e) Limiting oxygen index (LOI) testing results of Celgard, PVDF‐HFP, PEO, SSK‐20, and CQSE‐20. Burning behavior of f) SSK‐20, and g) CQSE‐20 during UL‐94 testing.

Differential scanning calorimetry (DSC) analysis reveals suppressed PVDF‐HFP crystallization behavior in SSK‐20 and CQSE‐20 (**Figure** **3**a). Compared to PVDF‐HFP, intermolecular hydrogen bonds in SSK‐20 lead to lower melting and cold crystallization temperatures, but the competitive anchoring of TFSI^−^ with UPy in CQSE‐20 leads to the destruction of the hydrogen bonds structure, so that the melting and cold crystallization temperatures remain essentially the same. LATP, UPyMA, PVDF‐HFP, SSKs, and SL‐CQSEs were tested for thermal stability using thermogravimetric analysis (TGA) (Figure 3b; Figure S14, Supporting Information). Concretely, LATP ceramic nanoparticles exhibit outstanding thermal stability, showing negligible mass loss up to 700 °C. UPyMA and PVDF‐HFP exhibit a single‐step thermal decomposition process, with initial decomposition temperatures (T_5%_) ≈200 and 420 °C, respectively, which are corresponding to the two‐step thermal decomposition process of SSKs. In terms of SL‐CQSEs, a four‐step thermal decomposition process is displayed, probably involving to carbonate, LiTFSI, copolymer, and PVDF‐HFP. According to the TGA result, the mass contents of LATP, PVDF‐HFP, LiTFSI, P(VC‐UPy), and FEC in CQSE‐20 can be obtained, that is 30.0%, 12.8%, 11.0%, 27.4%, and 18.8%, respectively (Figure S15, Supporting Information).

The mechanical properties of SSK‐20 and CQSE‐20 were evaluated using atomic force microscope (AFM) and tensile tests. The stress‐strain curves of SSKs and CQSE‐20 are illustrated in Figure 3c. The tensile strength values for PVDF‐HFP/LATP (SSK‐0), SSK‐15, SSK‐20, and CQSE‐20 are 251.3, 367.6, 487.4, and 332.4 kPa, respectively. While the elongation at break of SSKs remains relatively constant (≈5%), the tensile strength increases with the UPyMA content. For CQSE‐20, its elongation at break (47.0%) is nearly tenfold compared to SSK‐20, but accompanied by a decrease in tensile strength of ≈30%. In addition, Young's modulus of SSK‐20 and CQSE‐20 measured by AFM are 1.4 GPa and 270.2 MPa, respectively (Figure 3d). These AFM and tensile test results demonstrate the advantage of flexibility while retaining a certain strength for CQSE‐20.^[^ ^28^ ^]^

Limiting oxygen index (LOI) and vertical burning tests were conducted to assess the flame retardant properties of SSK‐20 and CQSE‐20. The LOI values of different materials are depicted in Figure 3e.^[^ ^29^ ^]^ Compared to the flammable Celgard PP, PVDF‐HFP, and PEO (LOI < 26%), the LOI values for SSK‐20 and CQSE‐20 are up to 31% and 47%, respectively, indicating that they are incombustible.^[^ ^30^ ^]^ Notably, the LOI value of CQSE‐20 is significantly higher than that of SSK‐20, indicating the superior fire safety of CQSE‐20 due to the formation of compact structure and the introduction of fluorine‐containing FEC in CQSE‐20. In the vertical burning tests, the ignition process involves two consecutive ignitions, each lasting 10 s. Both SSK‐20 and CQSE‐20 exhibit outstanding flame retardancy (Figure 3f,g). During the first ignition stage, SSK‐20 was ignited within 5 s and self‐extinguished within 10 s. In the second ignition, SSK‐20 was not ignited. Significantly, for CQSE‐20, it did not be ignited in neither the first nor second ignition stage.

### 2.2 Electrochemical Performance of SL‐CQSEs

**Figure 4:**
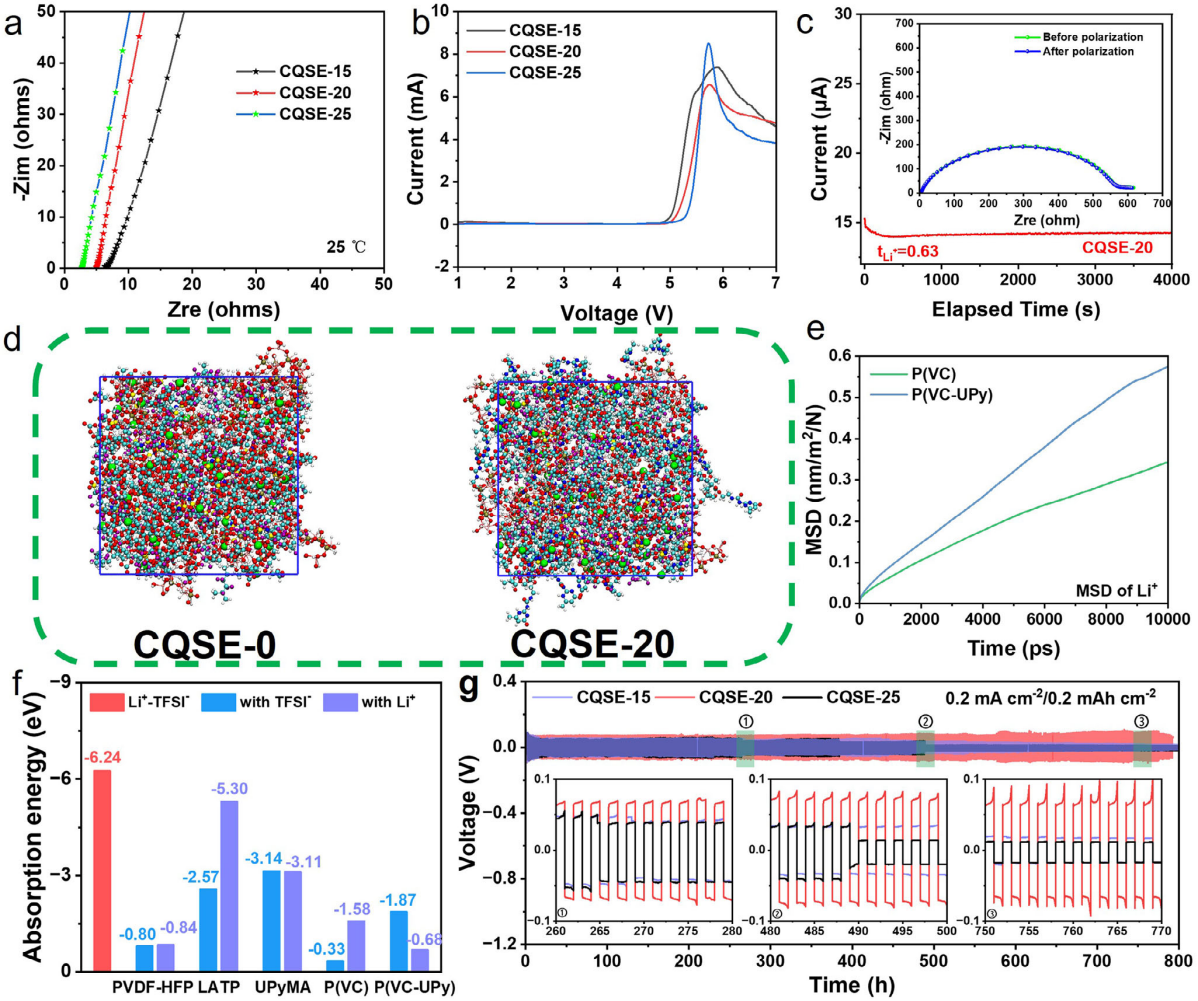
a) EIS curves of SS/SS cells with different SL‐CQSEs at 25 °C. b) LSV curves of Li/SS cells with different SL‐CQSEs at a sweep rate of 5 mV s^−1^ in a voltage range from 0 to 7 V. c) 10 mV polarization current‐time curve of the Li/CQSE‐20/Li cell and its Nyquist impedance spectra before and after polarization (inset). d) Snapshots of MD simulations of CQSE‐0 and CQSE‐20. e) Comparison of Li^+^ diffusion MSD. f) Absorption energies of different components for Li^+^ and TFSI^−^, respectively. g) Lithium stripping‐plating profiles of symmetric Li/Li cells with CQSE‐15, CQSE‐20, and CQSE‐25 at a current density of 0.2 mA cm^−2^ and a cycling capacity of 0.2 mAh cm^−2^.

The ionic conductivity of SL‐CQSEs was determined using EIS. The ionic conductivities of CQSE‐15, CQSE‐20, and CQSE‐25 at 25 °C were measured as 0.797, 1.06, and 2.86 mS cm^−1^, respectively (**Figure** **4**a). Notably, CQSE‐20 and CQSE‐25 exhibit outstanding ionic conductivity (>1 mS cm^−1^), being comparable to liquid electrolytes at 25 °C. The electrochemical stability of SL‐CQSEs was assessed through linear sweep voltammetry (LSV) measurements. CQSE‐15, CQSE‐20, and CQSE‐25 display electrochemical stability reaching 5.0, 5.1, and 5.3 V, respectively (Figure 4b). Li^+^ transference number (t_Li+_) as a crucial measure of SL‐CQSE performance represents the migration efficiency of Li^+^ in electrolytes. According to Equation S2 (Supporting Information), the calculated t_Li+_ values for CQSE‐15, CQSE‐20, and CQSE‐25 are 0.63, 0.63, and 0.66, respectively, surpassing the t_Li+_ value of traditional liquid electrolytes (0.38) (Figure 4c; Figure S16, Supporting Information).^[^ ^31^ ^]^ As the content of UPyMA increased, the t_Li+_ values also slightly increased, consistently remaining above 0.6, indicating the role of UPy units in anchoring anion movement.

Molecular dynamics (MD) simulating the Li^+^ diffusion pathways in CQSE‐0 and CQSE‐20, it is found that the copolymer P(VC‐UPy) in CQSE‐20 can release more free Li^+^ compared to the P(VC) in CQSE‐0 (Figure 4d). The abundant C═O groups in the copolymer can serve as transport sites for Li^+^, enhancing the Li^+^ transport rate. According to the calculation of the mean square displacement (MSD) of Li^+^ diffusion, the Li^+^ diffusion coefficient of P(VC‐UPy) is 5.6 × 10^−8^ cm^2^ s^−1^, while that of P(VC) is 4.8 × 10^−8^ cm^2^ s^−1^ (Figure 4e). Furthermore, the adsorption energies of PVDF‐HFP, LATP, and UPyMA for Li^+^ and TFSI^−^ were compared, respectively (Figure 4f). The adsorption energies of PVDF‐HFP and LATP for TFSI^−^ are smaller than that of them for Li^+^. Only adsorption energy of UPyMA for TFSI^−^ is larger than that of UPyMA for Li^+^, suggesting that UPyMA tends to anchoring the TFSI^−^ anion, which can result in the weakening of the intermolecular hydrogen bonds between UPyMA and PVDF‐HFP to a certain extent. This competitive effect could not only facilitate the Li^+^ migration, but also induce the formation of a stable SEI to inhibit SL‐CQSE/electrode interfacial side reactions and improve electrochemical stability. P(VC‐UPy) copolymer demonstrates stronger adsorption energy for the TFSI^−^ compared to P(VC), while exhibiting weaker adsorption energy for the Li^+^. This is conducive to increasing the Li^+^ concentration and enhancing the ionic conductivity. We then used NMR measurements to investigate how the anion TFSI^−^ interacts with the UPy unit to anchor TFSI^−^ (Figure S17, Supporting Information). With the introduction of UPyMA, the ^7^Li NMR characteristic peaks of UPyMA/LiTFSI remained basically the same, the ^19^F NMR characteristic peaks of UPyMA/LiTFSI appeared a downfield shift corresponding to the deshielding effect, indicating that the electron cloud density around the F atom of the TFSI^−^ anion decreased, and the ^1^H NMR characteristic peaks of UPyMA/LiTFSI appeared an upfield shift corresponding to the shielding effect, indicating that the electron cloud density around the H atom of the N─H bond of the UPy unit increased. This effect is attributed to the interaction between the highly electronegative F atoms of CF_3_ and positively charged fragments N−H groups of UPyMA. These improvements boost overall ionic conductivity, which is crucial for enhancing battery electrochemical performance.

Lithium stripping‐plating tests were conducted to assess the compatibility between the SL‐CQSEs and Li metal anode. The voltage profiles of Li/Li symmetric cells with CQSE‐0 and CQSE‐20 at 0.1 mA cm^−2^ cycling capacity of 0.1 mAh cm^−2^ are displayed in Figure S18 (Supporting Information). The overpotential of Li/CQSE‐0/Li cell gradually decreases from 82 to 62 mV and followed by a sharp drop to 29 mV after 8 h, indicating that the cell fails due to the side reaction between LATP and lithium metal. Then, the overpotential continually decreased to ≈0, reflecting a complete short circuit. Conversely, Li/CQSE‐20/Li cell exhibits a prolonged steady lithium stripping‐plating lifetime of over 700 h, maintaining the overpotential stably at ≈49 mV. The voltage profiles of the Li/Li symmetric cells with CQSE‐15, CQSE‐20, and CQSE‐25 at 0.2 mA cm^−2^ cycling capacity of 0.2 mAh cm^−2^ are displayed in Figure 4g. The overpotential of Li/CQSE‐15/Li cell consistently maintains at ≈56 mV for 270 h, then gradually decreases to 24 mV, indicating the occurrence of a short‐circuit in the cell. The overpotential of Li/CQSE‐25/Li cell dramatically drops to ≈24 mV after 490 h. In contrast, Li/CQSE‐20/Li cell displays a steady lithium stripping‐plating behavior with an overpotential of ≈80 mV over 750 h, demonstrating the good stability.

Furthermore, the surfaces of the cycled lithium metal anode from different Li/Li cells were analyzed (Figure S19, Supporting Information). The cycled lithium surface of CQSE‐15 and CQSE‐25 exhibits whisker‐like lithium formation, while CQSE‐20 results in a large area of flat morphology. For CQSE‐25, the stronger hydrogen bonds compared to CQSE‐20 would hinder the Li^+^ migration rate during long cycles, thereby inducing uneven Li^+^ deposition. These results suggest that CQSE‐20 presents the best stability for the Li metal anode because of the reasonable TFSI^−^ anchoring and hydrogen bonds competition, which is beneficial for the long‐term cycling stability of the lithium batteries.

### 2.3 Batteries Performance and Interface Characterization

**Figure 5:**
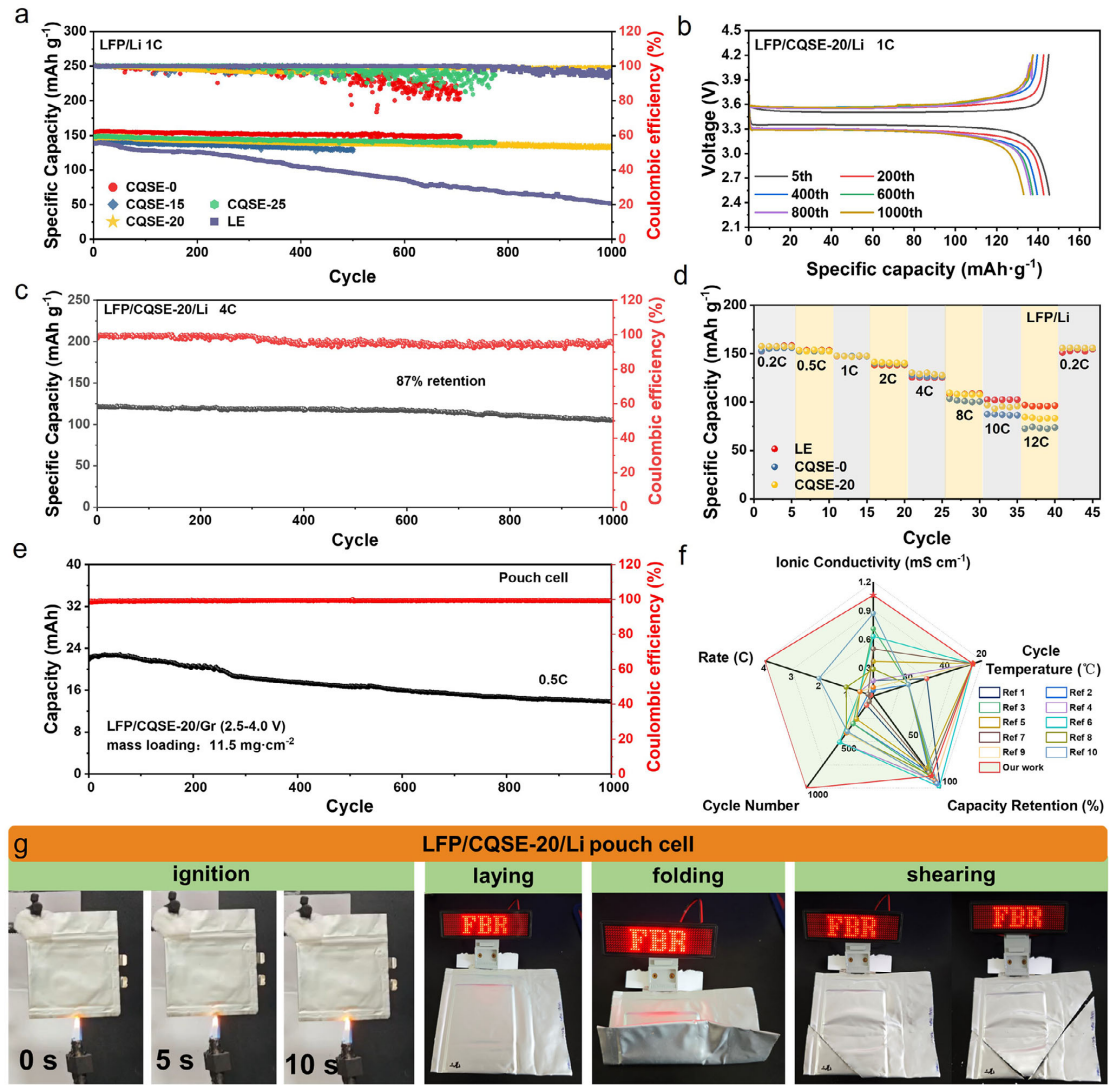
a) Room‐temperature cyclic performance of LFP/Li cells with CQSE‐0, CQSE‐15, CQSE‐20, CQSE‐25, and LE at 1C, respectively. b) 1C discharge‐charge profiles of the LFP/CQSE‐20/Li cell at different cycles. c) Room‐temperature cyclic performance of LFP/CQSE‐20/Li cell at 4C. d) Rate the performance of LFP/Li cells using different electrolytes. e) Room‐temperature cyclic performance of LFP/CQSE‐20/Gr pouch cell at 0.5C. f) Comparison of key performance parameters of CQSE‐20‐based cell with other electrolytes‐based cells (References were cited in Supporting Information). g) Photographs of the ignition test, and the lighting up LED lamp row test under different states at room temperature, for the LFP/CQSE‐20/Li pouch cell.

The room‐temperature cyclic performance of LFP/Li cells based on various SL‐CQSEs with different UPyMA contents was presented in **Figure** **5**a. The LFP/CQSE‐0/Li cell exhibits a gradual and fluctuant inconsistent decline in coulombic efficiency, dropping to 80% after 500 cycles. After introducing the UPyMA, for the LFP/CQSE‐15/Li cell, its coulombic efficiency is more stable and can maintain at 97% after 700 cycles, suggesting that P(VC‐UPy) copolymer induced the formation of stable SEI to ensure more high‐efficiency lithium stripping‐plating, albeit with failure observed after 500 cycles. With further increasing UPyMA content, for the LFP/CQSE‐20/Li cell, a significantly extended lifespan of over 1000 cycles is achieved at 1C, with an initial discharge capacity of 148.1 mAh g^−1^, a capacity retention of up to 90%, and a coulombic efficiency above 99%. Correspondingly, the LFP/CQSE‐20/Li cell capacity‐voltage curves under cyclic charging and discharging were recorded in Figure 5b. However, like the LFP/CQSE‐0/Li cell, the LFP/CQSE‐25/Li cell experiences severely fluctuant degradation in coulombic efficiency after 500 cycles, dropping as low as ≈80%, indicating deteriorative interfacial stability on lithium anode, in accord with the lithium stripping‐plating results. As for the LFP/Li cell with liquid electrolytes (LE), the long‐term cycling performance is poor, with a capacity retention of only 39% after 1000 cycles. Besides, we increased the LATP content in the SSK (PVDF‐HFP: LATP: UPyMA = 1:9:2) and found that during the phase separation process, the SSK curled up and did not remain flat due to the reduction of polymer components. We then assembled its LFP/Li cell to test the room‐temperature cyclic performance, and it can be found that the coulombic efficiency is unstable, indicating that increasing the LATP had leads to a poor compatibility with lithium metal (Figure S20, Supporting Information).

In addition, we investigate the high‐rate cycling capabilities of LFP/CQSE‐20/Li cell at 4C. At room temperature, the cell exhibits an initial discharge capacity of 122.0 mAh g^−1^, with a capacity retention of 87% over 1000 cycles (Figure 5c). The rate performance of the LFP/Li cells using LE, CQSE‐0, and CQSE‐20 was presented in Figure 5d. The LFP/CQSE‐20/Li cell demonstrates good rate performance even at high rate, delivering specific capacities of 157.5, 154.0, 146.8, 140.6, 130.4, 107.7, 95.4, and 83.8 mAh g^−1^ at 0.2C, 0.5C, 1C, 2C, 4C, 8C, 10C, and 12C, respectively. When the rate went back to 0.2C, a discharge capacity of 169.5 mAh g^−1^ was achieved. This result is superior to CQSE‐0 and comparable to liquid electrolytes (LE). The discharge/charge profiles at different rates also support the exceptional rate performance of the LFP/CQSE‐20/Li cell (Figure S21, Supporting Information). To verify whether the precursor solution is suitable for lithium metal and full cells, we assembled Li/Li and LFP/Li cells with the precursor solution. Lithium stripping‐plating test was conducted to assess the compatibility between the precursor solution and Li metal anode. The voltage profiles of Li/Li symmetric cell at 0.1 mA cm^−2^ cycling capacity of 0.1 mAh cm^−2^ are displayed in Figure S22a (Supporting Information). Li/Li cell exhibits a steady lithium stripping‐plating lifetime of 100 h, maintaining the overpotential stably at ≈24 mV. Besides, LFP/Li cell exhibits an initial discharge capacity of 145.6 mAh g^−1^, with a capacity retention of 90% over 20 cycles (Figure S22b, Supporting Information). These results indicate that this precursor solution is suitable for Li metal and full cells.

Furthermore, we assembled and tested pouch cells. LFP/CQSE‐20/Gr pouch cell exhibits an initial discharge capacity of 22.8 mAh at 0.5C, with a capacity retention of 60.7% over 1000 cycles (Figure 5e). Additionally, the LFP/CQSE‐20/Li pouch cell delivers an initial discharge capacity of 142.0 mAh g^−1^ at 0.1C, dropping slightly to 131.5 mAh g^−1^ at 0.5C, and can stably run for nearly 30 cycles (Figure S23, Supporting Information). A comparison of the essential characteristics of CQSE‐20 with some LATP‐based electrolytes previously reported was conducted (Figure 5f; Table S1, Supporting Information). This comparison highlights the ionic conductivity, rate performance, cycling lifespan, and operating temperature. It has been found that the CQSE‐20 endows lithium batteries with comprehensive advantages.

We then evaluated the fire safety of the LFP/CQSE‐20/Li pouch cell through ignition tests. The pouch cell was incombustible and even did not expand under the burning, proving that it has good fire safety (Figure 5g). In addition, we tested the use of the pouch cell under different destructive conditions and found that the pouch cell can light up the LED lamp row regardless of folding and shearing. The above results show that the LFP/CQSE‐20/Li pouch cell exhibits good fire safety and resistance to mechanical abuse.

Subsequently, the morphology of the lithium metal anode was observed by SEM. The fresh Li foil surface is smooth and flat (Figure S24, Supporting Information). After 1000 cycles at 1C, we disassembled the LFP/LE/Li cell and the LFP/CQSE‐20/Li cell to observe the lithium anode surface. The Li anode surface from the LFP/LE/Li cell appears rough, loose, and shows abundant holes, “dead Li” and dendrites due to excessive side reactions and serious electrochemical polarization. In contrast, the Li anode surface from the LFP/CQSE‐20/Li cell is compact and smooth, indicating that the CQSE‐20 effectively suppresses the formation of lithium dendrites, and promotes uniform Li deposition (Figure S25, Supporting Information). In addition, we observe the lithium anode surface after 1000 cycles at 4C. The Li anode surface is also flat and uniform, indicating the compatibility of CQSE‐20 with lithium metal at a high rate and long cycle (Figure S26, Supporting Information).

**Figure 6:**
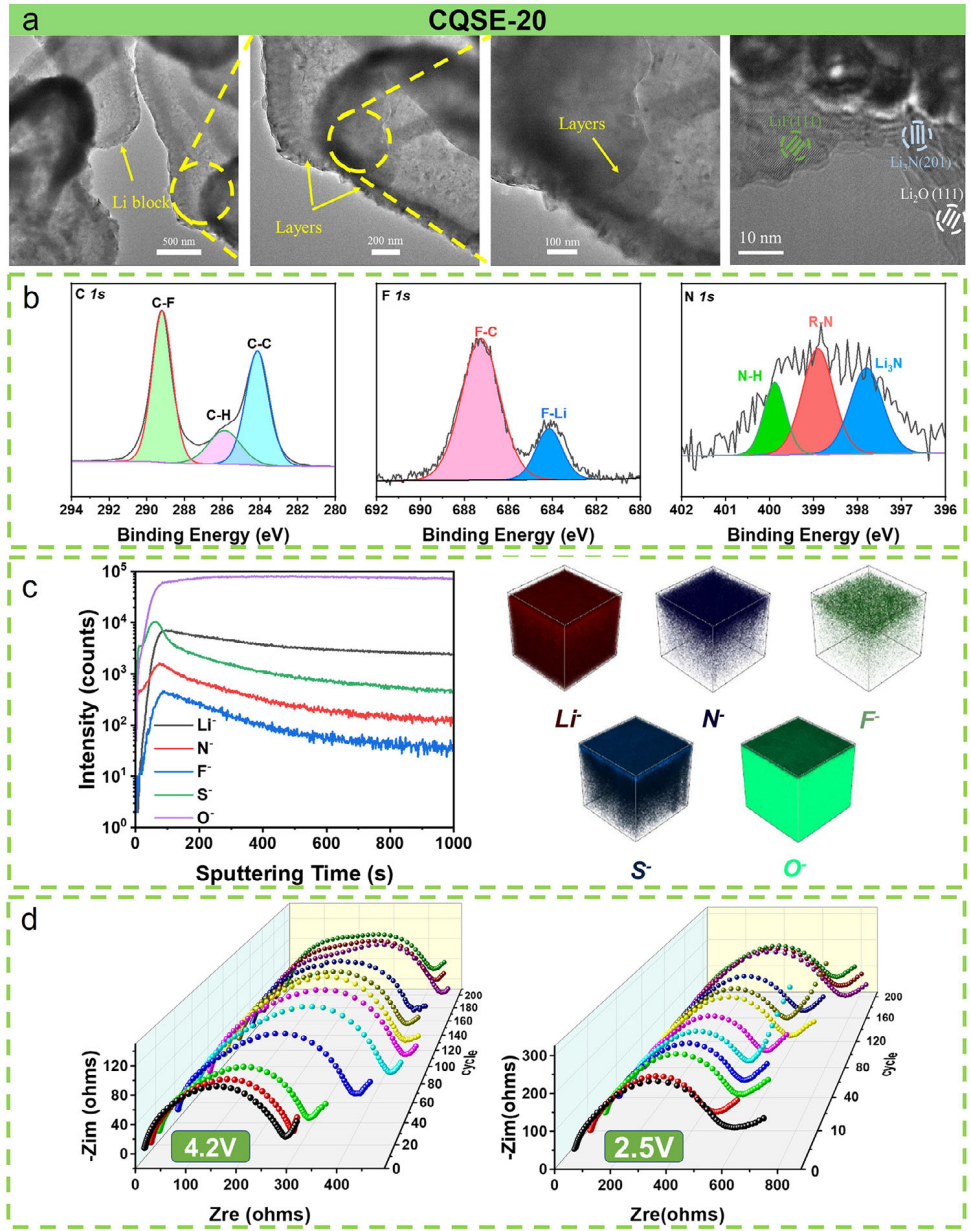
a) Cryo‐TEM of Li deposition morphology and SEI structure using CQSE‐20. b)The XPS curves and c) TOF‐SIMS depth profiles of the cycled Li metal anode after 1000 cycles at 1C from the LFP/CQSE‐20/Li cell. d)The Nyquist plots of LFP/CQSE‐20/Li cell were measured at 4.2 and 2.5 V with different cycles at 1C.

Cryogenic transmission electron microscopy (Cryo‐TEM) was utilized to analyze the structure of SEI layer. Samples were prepared following the method outlined in reference,^[^ ^32^ ^]^ where Li was directly deposited onto the Cu TEM grid within a 2032 coin cell. Deposited Li for the LFP/LE/Li cell displays a dispersed island‐like structure with numerous voids (Figure S27, Supporting Information). The SEI contains Li_2_O (111) and Li_2_CO_3_ (002). Deposited Li from the LFP/CQSE‐20/Li cell exhibits a layered, wafer‐like structure without voids (**Figure** **6**a), and there are LiF (111), Li_3_N (201), and Li_2_O (111) in its SEI. This Li_3_N/LiF‐rich inorganic and organic hybrid SEI contributes to rapid Li^+^ transport, so as to effectively promote uniform Li deposition.^[^ ^33^ ^]^

X‐ray photoelectron spectra (XPS) and time‐of‐flight secondary ion mass spectrometry (TOF‐SIMS) were adopted to detect the chemical composition of the SEI on the Li surface from the LFP/CQSE‐20/Li cell after 1000 cycles at 1C. The C *1s*, F *1s*, and N *1s* spectra of the cycled Li surface are displayed in Figure 6b. The peaks of C‐F (289.2 eV), C‐H (285.9 eV), and C‐C (284.1 eV) in the C *1s* spectra, as well as C‐F (687.3 eV) and LiF (684.2 eV) in the F *1s* spectra, are attributed to the decomposition of PVDF‐HFP and FEC. N‐H (400.3 eV), R‐N (399.1 eV), and Li_3_N (397.6 eV) peaks in the N *1s* spectra are attributed to the decomposition of UPy and TFSI^−^. The XPS result of cycled Li surface after 1000 cycles at 4C was consistent with the results after 1C cycling (Figure S28, Supporting Information). TOF‐SIMS depth profiles of the cycled Li surface reveal that the intensities of N^−^, F^−^, and S^−^ signals increase at first, and then decrease slowly as reaching the peak values. The O^−^ and Li^−^ signals intensities rapidly rise at the beginning, and gradually reach to stable values after the sputtering times of ≈100 s. The 3D views depict the spatial distribution of Li^−^, N^−^, F^−^, S^−^, and O^−^ signals on the Li metal surface with depth, confirming the presence of an inorganic/organic hybrid SEI layer (Figure 6c). These results align with Cryo‐TEM and XPS, demonstrating UPy and anion‐induced Li_3_N/LiF‐rich hybrid SEI.

To investigate the role of CQSE‐20 in stabilizing interfaces in LFP/Li cells during cycling, we conducted the EIS measurements at charge (4.2 V) and discharge (2.5 V) states at different cycle numbers (Figure 6d). The bulk resistance (R_b_) at high frequencies remains unchanged over 200 cycles during both charging at 4.2 V and discharging at 2.5 V. However, the interfacial resistance at medium frequencies displays distinct evolution over 140 cycles at 4.2 and 2.5 V. The fitted interface impedance values for the LFP/CQSE‐20/Li cell at both voltage states are displayed in Figure S29 (Supporting Information). During the charge, the interface impedance steadily increased from ≈250 Ω (20 cycles) to ≈400 Ω (60 cycles). This trend is attributed to the gradual formation of the SEI on the lithium anode during the initial 60 cycles. In contrast, during the discharge, the interface impedance fluctuated between 500 and 600 Ω, showing a slow upward and gradually stabilizing ≈600 Ω (200 cycles). We then disassembled the LFP/CQSE‐20/Li cell after 200 cycles at 1C and analyzed the chemical structure changes of CQSE‐20 by FT‐IR (Figure S30, Supporting Information). The symmetrical stretching peak of C‐F experiences a blue shift at 4.2 and 2.5 V, derived from the anchoring of the UPy unit with the TFSI^−^, which leads to an increase in the electron cloud density of the C─F bond. Subsequently, the morphology of the cycled CQSE‐20 from LFP/CQSE‐20/Li cell after 1000 cycles at 1C was observed by SEM (Figure S31, Supporting Information). The cycled CQSE‐20 surface is dense and smooth, and its cross‐section SEM image shows that the fibrous and porous structures are intact, without obvious cracks and lithium dendrites.

## 3 Conclusion

In summary, the SL‐CQSEs for lithium metal batteries were prepared based on a intercomponent multiscale coupling strategy, in which self‐assembly nanofibers, PVDF‐HFP, and in situ formed copolymer provide rigid‐flexible framework, UPy units anchor TFSI^−^ anions and form intermolecular hydrogen bonds with PVDF‐HFP, and LATP and copolymer participate in the Li^+^ transport. TFSI^−^ anchoring and intermolecular hydrogen bonding competition reconciles the conflict of electrochemical performance and other properties. As a result, CQSE‐20 is completely non‐combustible passing UL‐94 V‐0 rating with a LOI of 47%, and achieves a high ionic conductivity of 1.06 mS cm^−1^ at 25 °C, a t_Li+_ of 0.63, and a wide electrochemical window of 5.1 V. In addition, anion‐induced Li_3_N/LiF‐rich hybrid SEI ensures uniform Li^+^ flux and stable interfacial integrity, enabling stable cycling for over 700 h at 0.1 and 0.2 mA cm^−2^. Significantly, LFP/CQSE‐20/Li cells exhibit exceptional high‐rate performance up to 12C and room‐temperature long lifespan with initial discharge capacity of 148.1 mAh g^−1^ and capacity retention of 90% after 1000 cycles at 1C. Even at a 4C high rate, it still delivers an initial discharge capacity of 122.0 mAh g^−1^ with 87% retention after 1000 cycles. Beyond these, LFP/Gr and LFP/Li pouch cells using CQSE‐20 also show good cycling performance and the safety of fire and mechanical damages, demonstrating the potential for safer high‐performance lithium metal batteries.

## Conflict of Interest

The authors declare no conflict of interest.

## Supporting information



Supporting Information

## Data Availability

The data that support the findings of this study are available from the corresponding author upon reasonable request.
