# Supplementary material for: Competitive Anion Anchoring and Hydrogen Bonding in Multiscale‐Coupling Composite Quasi‐Solid Electrolytes for Fire‐Safety and Long‐Life Lithium Metal Batteries
Source: Adv Sci (Weinh). 2025 Mar 24;12(19):2501012. doi: 10.1002/advs.202501012 (PMC12097025; doi:10.1002/advs.202501012)
Supplement: Supplementary file 1 — Supporting Information [file ADVS-12-2501012-s001.docx]

Supporting Information

**Competitive Anion Anchoring and Hydrogen Bonding in Multiscale-Coupling Composite Quasi-Solid Electrolytes for Fire-Safety and Long-Life Lithium Metal Batteries**

Ding Hu, Guo-Rui Zhu, Ping-Hui Duan, Si-Chong Chen*, Gang Wu*, Yu-Zhong Wang

The Collaborative Innovation Center for Eco-Friendly and Fire-Safety Polymeric Materials (MoE), National Engineering Laboratory of Eco-Friendly Polymeric Materials (Sichuan), State Key Laboratory of Polymer Materials Engineering, College of Chemistry, Sichuan University, Chengdu 610064, Sichuan, China
E-mail: gangwu@scu.edu.cn, chensichong@scu.edu.cn

**1. Experimental section**

**1.1. Chemicals：** LATP powder (D50=600 nm) were received from Shenzhen Kejing Star Tech Co. Ltd. Lithium bis(trifluoromethanesulfonic)imide (LiTFSI, 99%), vinylene carbonate (VC, 98%), fluoroethylene carbonate (FEC, 98%), poly(vinylidene fluoride-co-hexafluoropropylene) (PVDF-HFP, average Mw ~455,000, average Mn ~110,000), 2-amino-4-hydroxy-6-methyl pyrimidine, 2-isocyanatoethyl methacrylate, dimethyl sulfoxide (DMSO, >99.8%), N-methyl-2-pyrrolidone (NMP, >99.5%), 2,2'-azobis(2-methylpropionitrile) (AIBN, 99%) were received from Aladdin. The commercial liquid carbonate electrolytes (LE, 1 M LiPF_6_ in EC/DEC/DMC, 1:1:1 by volume ratio), and LiFePO_4_ (LFP) were bought from Guangdong Canrd New Energy Technology Co., Ltd.

**1.2. Synthesis of the UPyMA** ^[1]^：

The preparation of the UPyMA is shown in Figure S1. Briefly, 2-amino-4-hydroxy-6-methyl pyrimidine (4.0 g), anhydrous DMSO (50 mL) in a dried glass vessel equipped with magnetic stirring was heated to 150℃. The heat treatment was allowed to proceed for 10 min. Subsequently, 2-isocyanatoethyl methacrylate (5.5 g) was added to the glass vessel and then stirred at room temperature. The precipitated product was collected by vacuum filtration, washed several times with acetone to remove residual DMSO, and dried under a vacuum oven at 45℃ for 6 h. The UPyMA monomer was characterized by ^1^H and ^13^C NMR.

**1.3.** **Preparation of SSKs**:

The flexible SSKs were prepared via blade coating and phase separation process. Firstly, 0.3 g PVDF-HFP was dissolved in N-methyl-2-pyrrolidone, then LATP powder (0.7 g) was added and mixed. Lastly, different mass UPyMA (15 wt%, 20 wt%, 25 wt% of PVDF-HFP and LATP mixture) was added and stirred at ambient temperature for 6 h to form the homogenous slurry. Subsequently, the slurry is loaded onto the glass plate through the use of a scraper, and then rapidly immersed in deionized water that lasts 30 min. The SSKs were collected and dried under vacuum at 60℃ for 12 h to remove residual solvent.

**1.4. Preparation of SL-CQSEs:**

The SL-CQSEs were prepared by in-situ polymerization of the precursor solution. The precursor solution was prepared as follows: VC and FEC with a 1:1 volume ratio were firstly mixed, then 1 M LiTFSI and AIBN (0.2 wt% of VC) were added and dissolved to form a homogeneous solution. The above precursor solution (50 μL) was injected into an SSK, and assembled in a 2032 coin cell under a pressure of 750 kg, the encapsulated cell subsequently was heated at 60℃, and standing for 10 h.

**2. Materials characterization:**

Nuclear magnetic resonance (NMR) spectra of all samples were performed on the NMR instrument (Bruker AV II 400MHz, DMSO-d_6_) using tetramethylsilane (TMS) as a reference. All samples' Fourier transform infrared (FTIR) spectra were performed on a Nicolet (Thermo Fisher, iN10) FTIR instrument at room temperature. The microstructure morphology of the SSK and lithium metal was observed by field emission scanning electron microscopy (FE-SEM, JEOL JSM-7500F). The crystallinity of LATP powder, the SSK was characterized by X'Pert Pro MPD X-ray diffraction (XRD). XPS (Thermo Scientific K-Alpha+) with a monochromatic Al Kα radiation was used to analyze the chemical composition of lithium metal. Thermogravimetric analysis (TGA) was performed by using a DSC2500 thermo-analyzer instrument (TA) under N_2_ flow at a heating rate of 10 °C min^−1^. A differential Scanning Calorimeter (DSC) was performed by using a TGA5000 thermo-analyzer instrument (TA) under N_2_ flow. Underwriter Laboratory 94 vertical burning tests (UL-94 V) were performed on a CZF-2 instrument with a dimensional size of 80×10×1 mm^3^. Limiting oxygen index (LOI) tests were examined on an oxygen index flammability gauge (JF-3, JNFX Co., Ltd) according to ASTM D2863-77 standard with a dimensional size of 80×10×1 mm^3^. The Young’s modulus and surface microtopography of the SSK and SL-CQSE were performed by AFM (Bruker Dimension Icon). Time of flight secondary ion mass spectrometry (TOF-SIMS, tofsims5, Münster, Germany) was used to analyze the 3D components distribution of cycled lithium metal anode interface. For cryo-TEM measurement, Li was directly deposited on the Cu grid (200 mesh) at a current density of 0.5 mA cm^-2^ and a cycling capacity of 0.5 mA cm^-2^. After immersing in dimethyl carbonate overnight to remove residual Li salt, the Li-deposited Cu grid was transferred into a cryogenic vessel containing liquid nitrogen and then transferred into TEM for the test (FEI Talos Arctica).

**3. Cell Assembly:**

The LFP positive cathode was prepared as follows: the LFP active materials, Super P, and PVDF (HSV 900) (8:1:1 by weight) are mixed in NMP solvent to form the homogenous slurry, subsequently cast the slurry on an Al foil by doctor-blade followed by the oven and vacuum drying at 80℃ for 12 h. The active materials loading of the cathode was about 1.6 mg cm^-2^. For the LFP cathode of the pouch cell, we purchased a high-loading LFP cathode (11.5 mg/cm^2^) from Guangdong Canrd New Energy Technology Co., Ltd and used it directly. The Li foils 15.6 mm in diameter were directly used as lithium anodes.

CR2032 coin-typed cells were assembled and disassembled in an argon-filled glovebox. To assemble the LFP/SL-CQSE/Li cell, the SSK was first put on one LFP cathode plate, then 50 µL precursor solution was injected into SSK. Afterward, a lithium anode was put on top of the SSK containing the precursor solution. Finally, a steel disc was put on the lithium anode, and the cell was sealed to finish the cell assembly under a pressure of 750 kg. Similar procedures were performed to assemble the SS/SL-CQSE/SS, SS/SL-CQSE/Li, and Li/SL-CQSE/Li cells. The galvanostatic charge/discharge experiments were tested in the voltage range of 2.5-4.2 V using a galvanostatic test instrument (Shenzhen Neware Electronics Co., Ltd) at room temperature.

**4. Electrochemical characterization:**

Electrochemical impedance spectroscopy (EIS) was used to analyze the interfacial impedance with a multi-channel electrochemical station (ParSTAT MC). The parameter was set in the frequency range of 1 MHz to 0.1 Hz to analyze. The ionic conductivity of SL-CQSE was calculated based on the EIS (frequency range: from 1 MHz to 0.1 Hz, AC amplitude:10 mV) results and the following Equation S1:

$$\text{σ=}\frac{\text{L}}{\text{R×S}}\text{ }\text{\#}\text{Equation}\text{ S}\text{1}$$

Where L represents the thickness of SL-CQSE, R, and S represent the bulk resistance and effective contact area of SL-CQSE with stainless steel electrodes. Based on the Li/SL-CQSE/SS cell, the electrochemical window was calculated by linear sweep voltammetry (LSV) at a sweep rate of 5 mV s^-1^ with a voltage range from 0 V to 7 V.

The Li/SL-CQSE/Li cell was employed to measure the Li^+^ transference number (t_Li+_) based on the AC impedance and the DC polarization. This was calculated using Equation S2.

$$\text{ }\text{t}_{\text{Li}^{\text{+}}}\text{=}\frac{\text{I}_{\text{ss}}\left( \text{ΔV-}\text{I}_{\text{0}}\text{R}_{\text{0}} \right)}{\text{I}_{\text{0}}\left( \text{ΔV-}\text{I}_{\text{ss}}\text{R}_{\text{ss}} \right)}\text{ \#}\text{Equation}\text{ S}\text{2}$$

Where I_0_ and I_ss_ represent the initial and steady current. R_0_ and R_ss_ represent the initial and steady resistance before and after polarization. ΔV represents the oscillation voltage (10 mV).

**5. Computational Methods:**

The first-principles density functional theory calculations were carried out using the Cambridge Sequential Total Energy Package (CASTEP) package. The electron exchange function was calculated using Perdew-Burke Brinkerhoff (PBE) described by generalized gradient approximation (GGA). The C.G.BroydenC-R.Fletcher-D.Goldfarb-D.F.Shanno (BFGS) was selected for the minimization algorithm.

The molecular dynamics were performed using the software package GROMACS. The atomic interactions were parameterized by the generation amber force field (GAFF), and RESP2 charge obtained from Multiwfn was applied in the calculations. The MSD of the Li^+^ was analyzed by the toolkits of GROMACS.


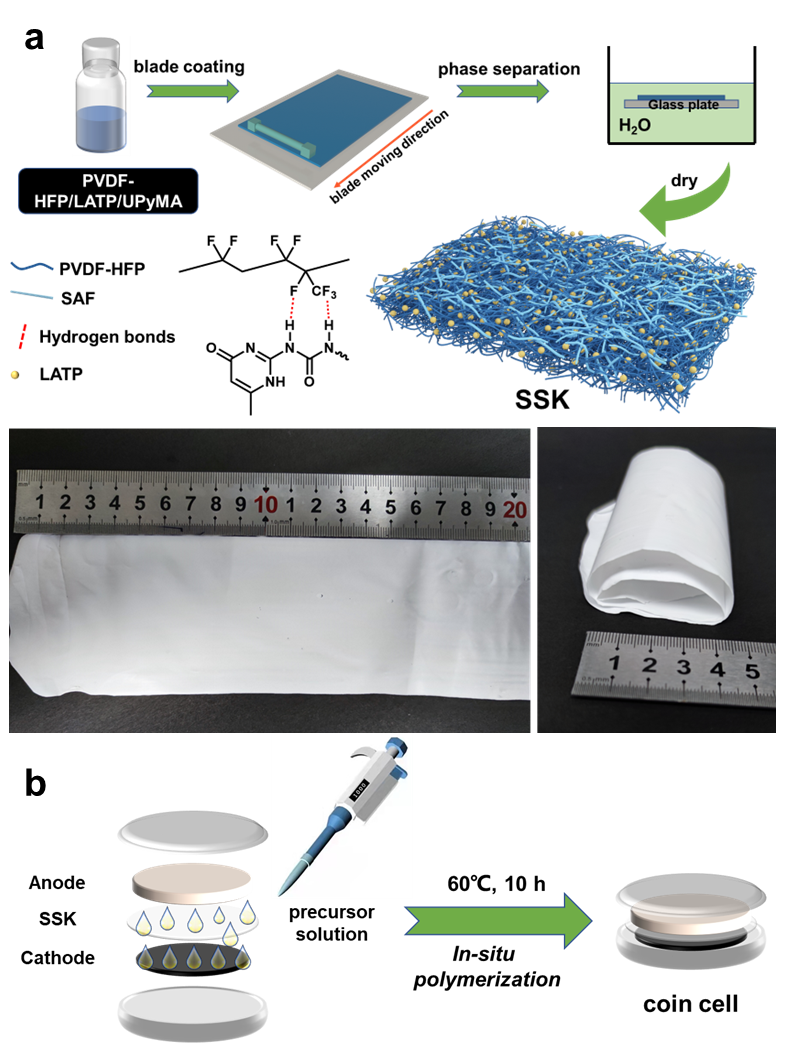


**Figure S1.** Fabrication flowchart of SL-CQSE. (a) internal forces and photograph of the SSK; (b) in-situ polymerization.

**Figure S2.** Synthesis of UPyMA monomer.


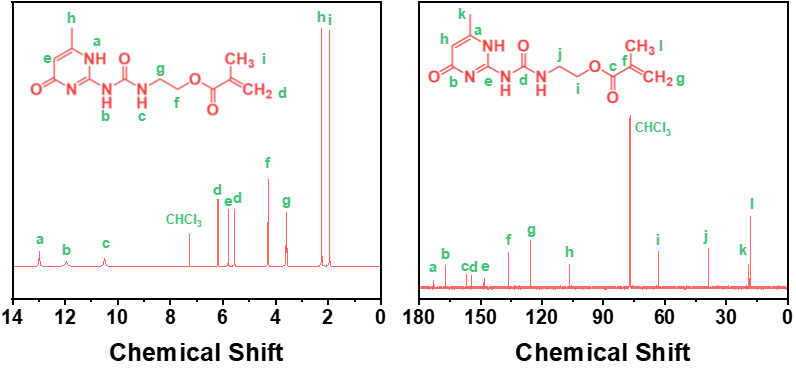


**Figure S3.** ^1^H and ^13^C NMR spectra of the UPyMA monomer in CDCl_3_.


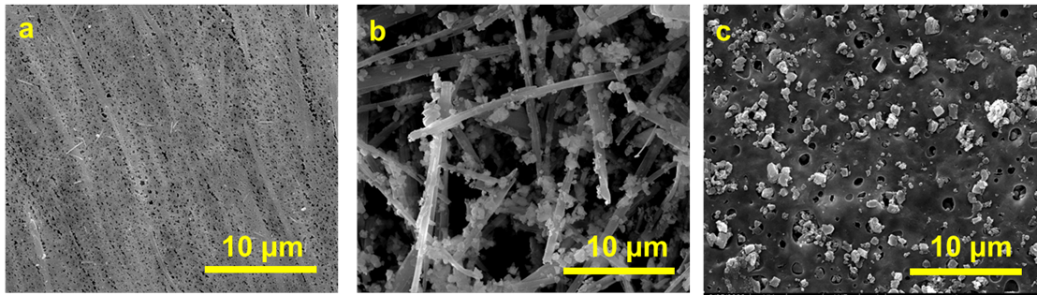


**Figure S4.** Top-view SEM images of (a) PVDF-HFP/UPyMA, (b) LATP/UPyMA, (c) PVDF-HFP/LATP.


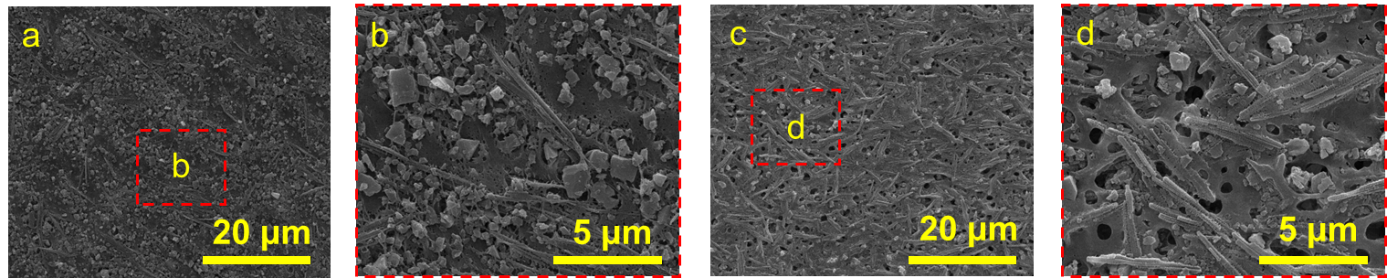


**Figure S5.** Top-view SEM images of (a, b) SSK-15; (c, d) SSK-25.

**
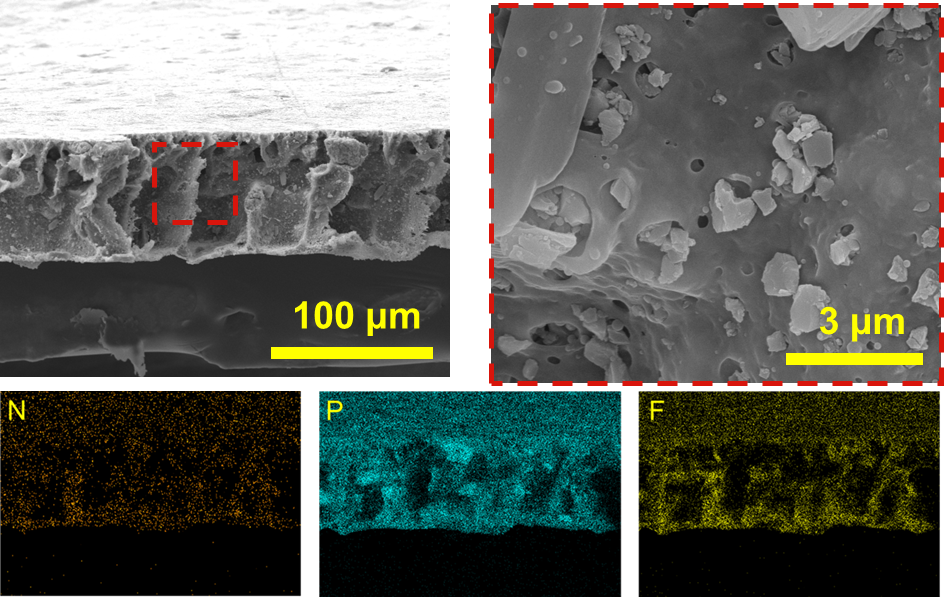
**

**Figure S6.** Side-view SEM images of the SSK-20 and the energy-dispersive X-ray spectroscopy (EDS) mapping images of N, P, and F elements in SSK-20.


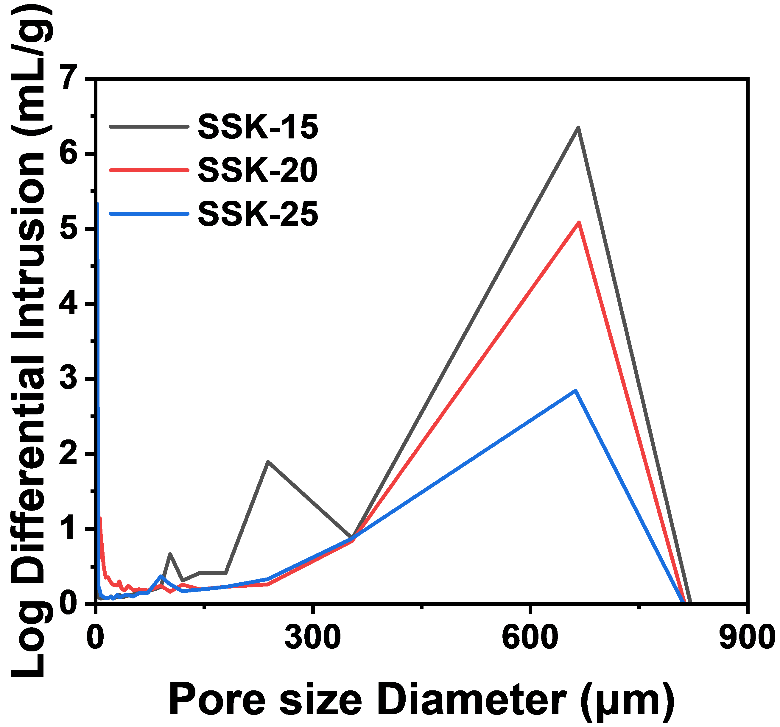


**Figure S7.** The pore size distribution profiles of SSK-15,20,25.


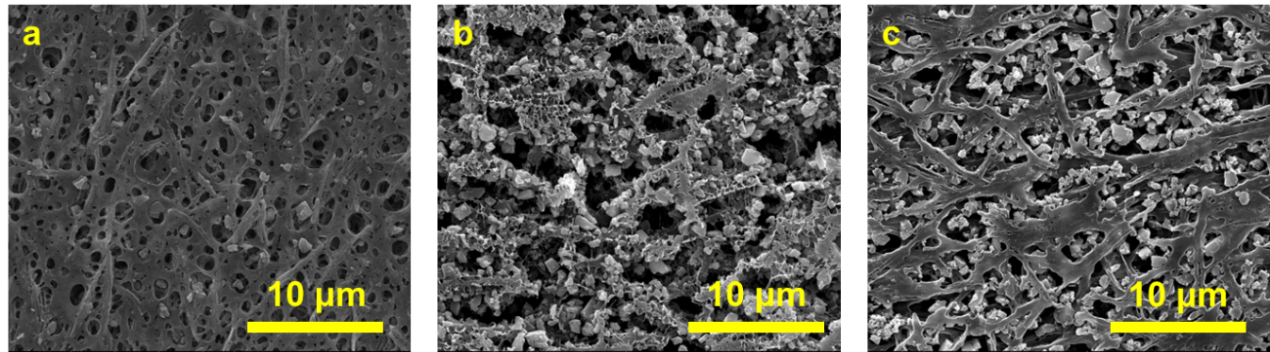


**Figure S8.**  Top-view SEM images of the SSK-20 by phase separation with different solvents: (a) H_2_O, (b) EtOH, (c) H_2_O: EtOH (1:1 by volume ratio).


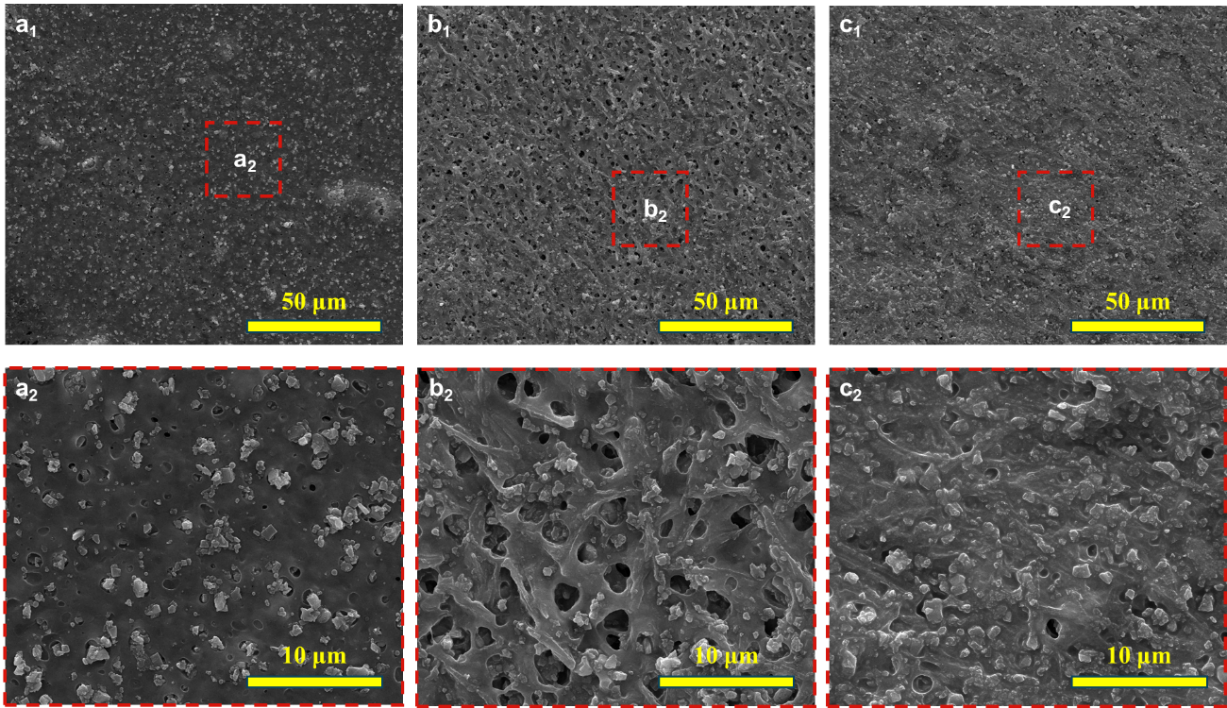


**Figure S9.** Top-view SEM images of the SL-CQSEs. (a) CQSE-0; (b) CQSE-15; (c) CQSE-25.


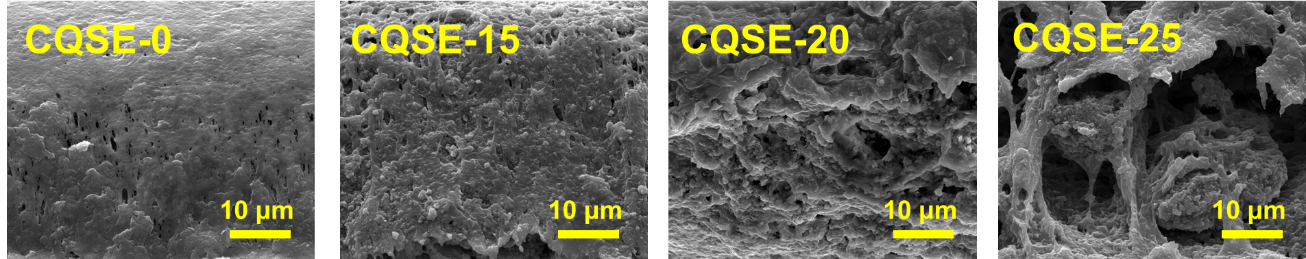


**Figure S10.** Cross-section SEM images of the CQSE-0, CQSE-15, CQSE-20, CQSE-25.


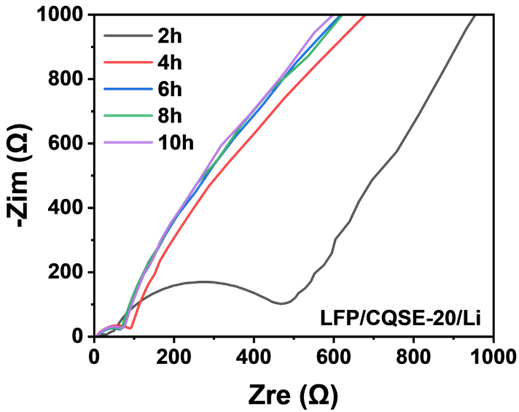


**Figure S11.** EIS curves of LFP/CQSE-20/Li at different heating times at 60℃.


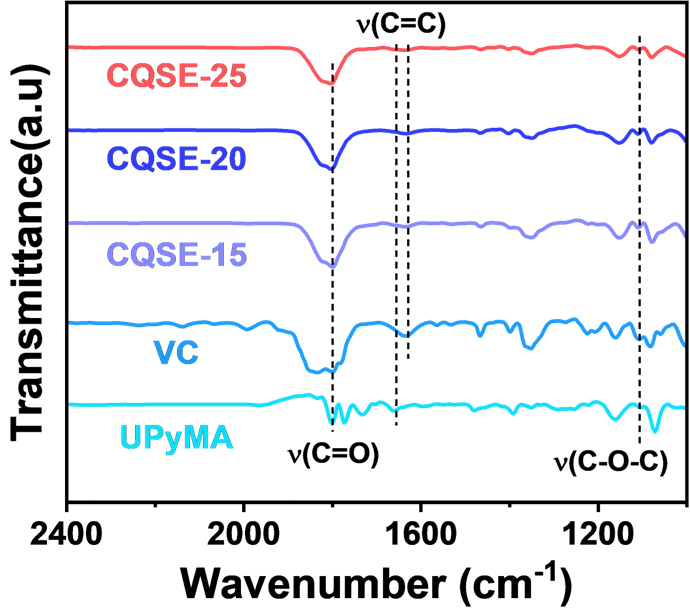


**Figure S12.** FT-IR spectra of UPyMA, VC, CQSE-15, CQSE-20, CQSE-25.


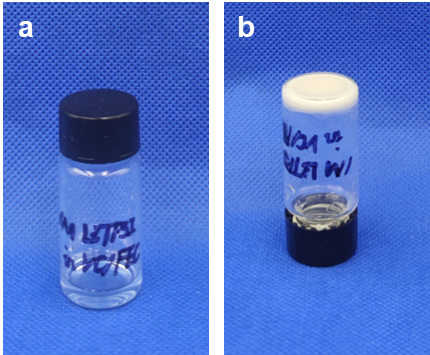


**Figure S13.** Optical photograph of the precursor solution before and after polymerization at 60 ℃ for 10 h.


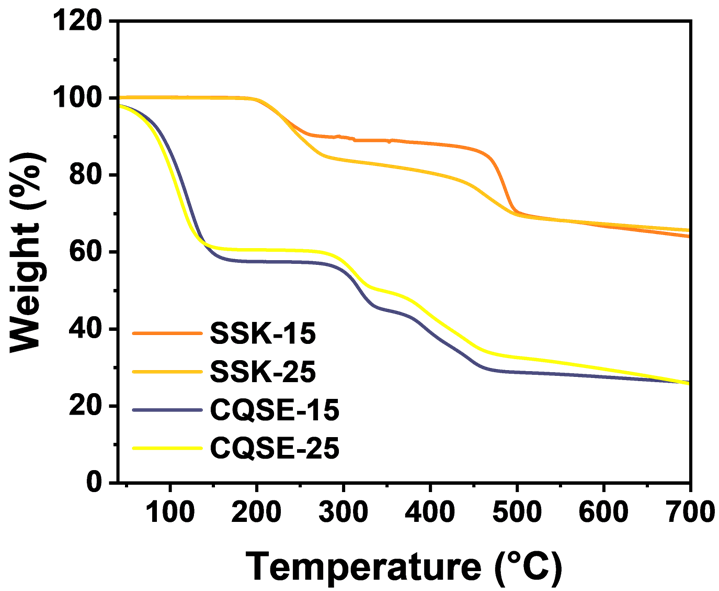


**Figure S14.** TGA spectra of the SSK-15, SSK-25, and CQSE-15, CQSE-25.


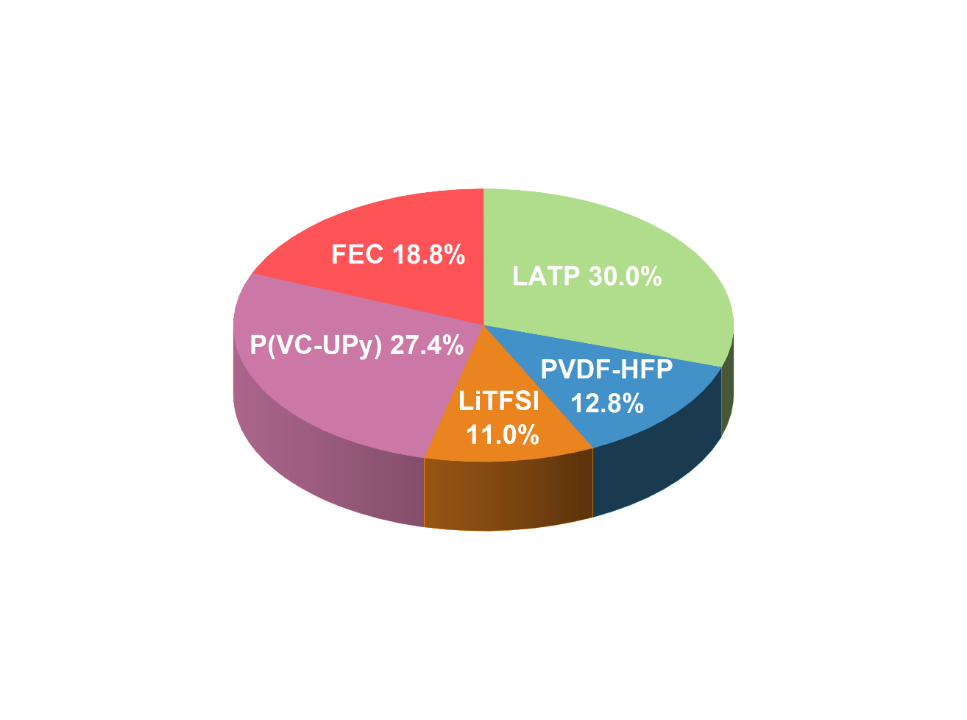


**Figure S15.** Quantification results of each component in CQSE-20.


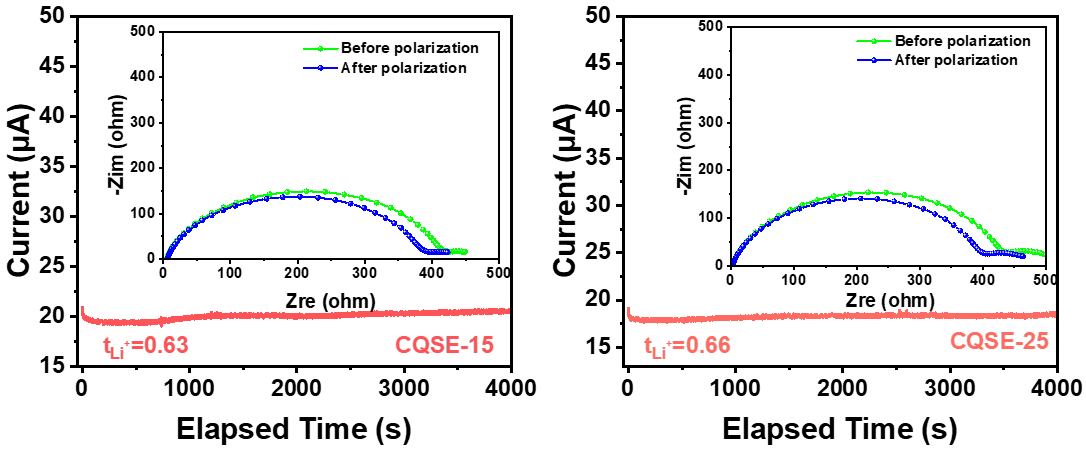


**Figure S16.** 10 mV polarization current-time curve of the Li/Li symmetric batteries using CQSE-15, CQSE-25 and Nyquist impedance curves before and after polarization (inset).

**
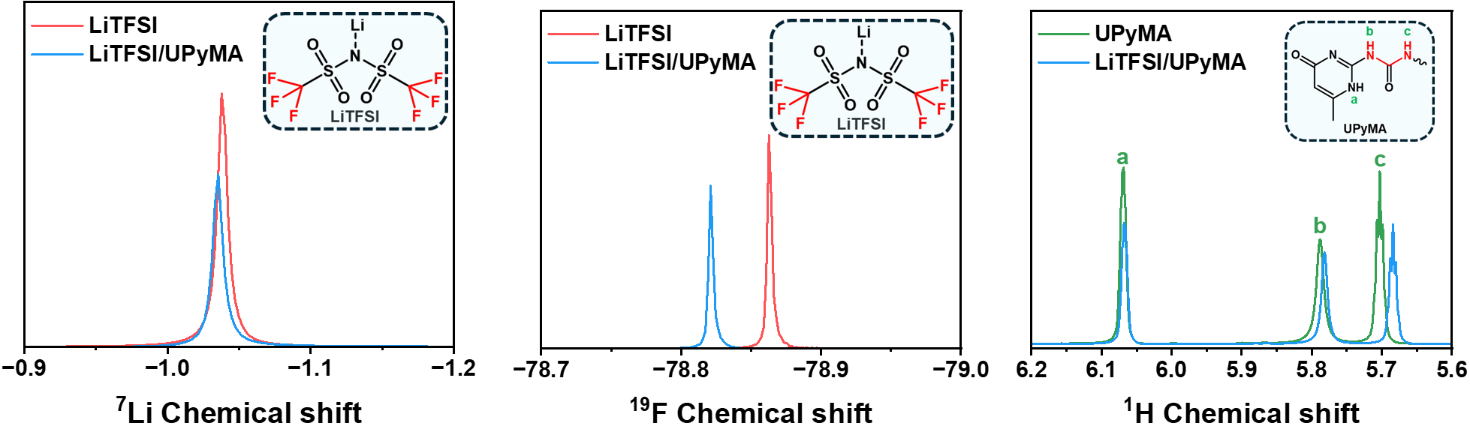
**

**Figure S17**. NMR spectra of LiTFSI, UPyMA and UPyMA/LiTFSI.


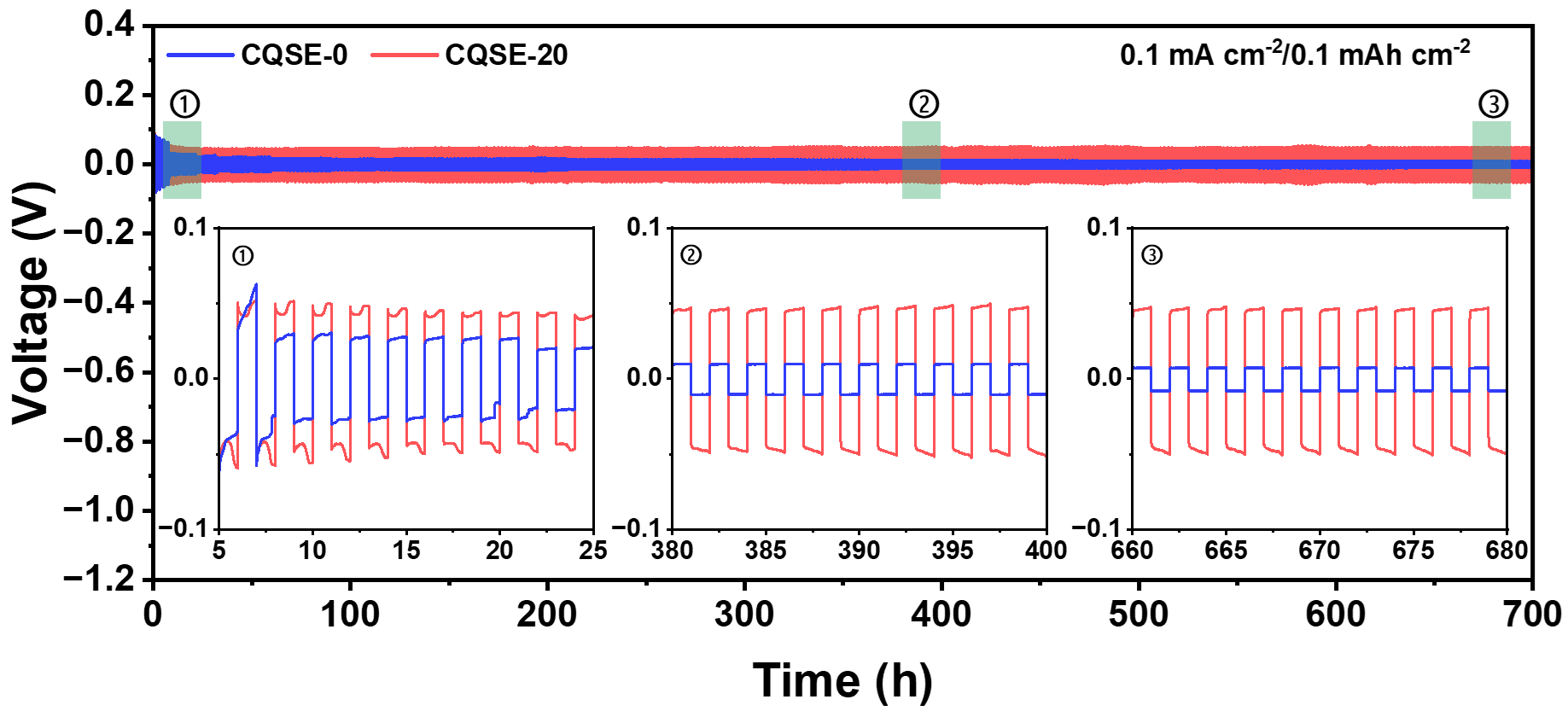


**Figure S18.** Lithium stripping-plating profiles of Li/Li cells with CQSE-0, CQSE-20 at a current density of 0.1 mA cm^−2^ with a cycling capacity of 0.1 mAh cm^−2^.


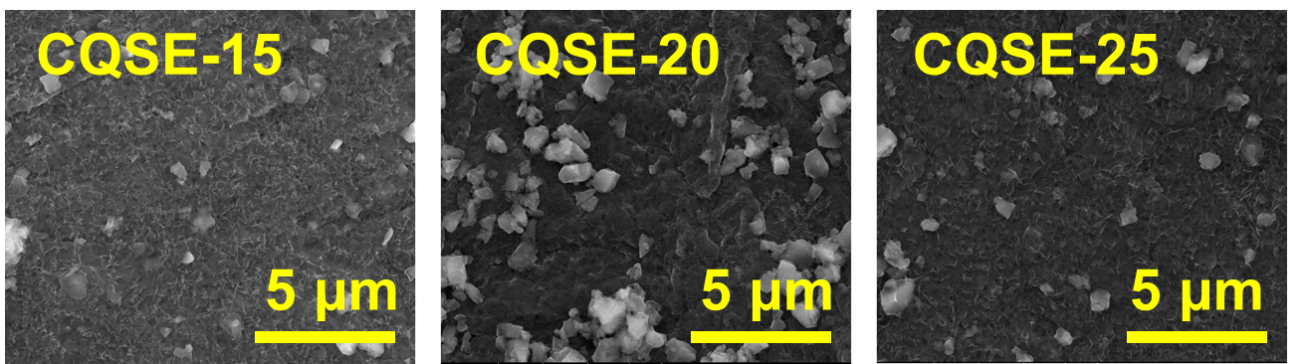


**Figure S19.** Top-view SEM images of deposited lithium surface after 800 h using different SL-CQSEs (Li/Li).

**
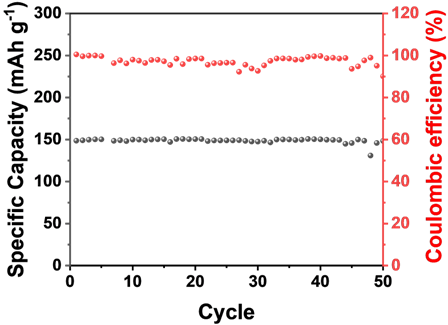
**

**Figure S20.** Room-temperature cyclic performance of LFP/Li cell based on the CQSE with an increased LATP content (PVDF-HFP: LATP: UPyMA = 1:9:2 in the SSK) at 1C.


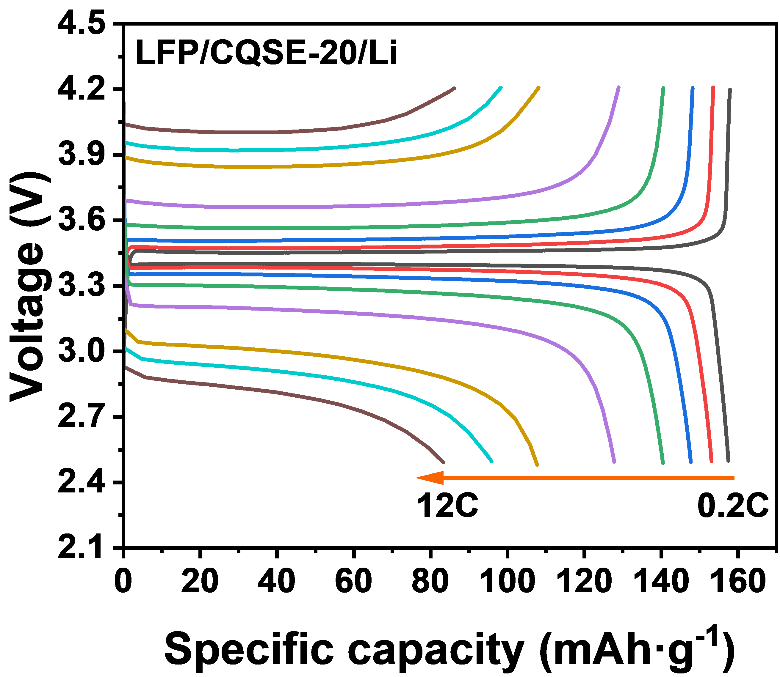


**Figure S21.** Discharge/charge profiles of the LFP/CQSE-20/Li cells at various rates.

**
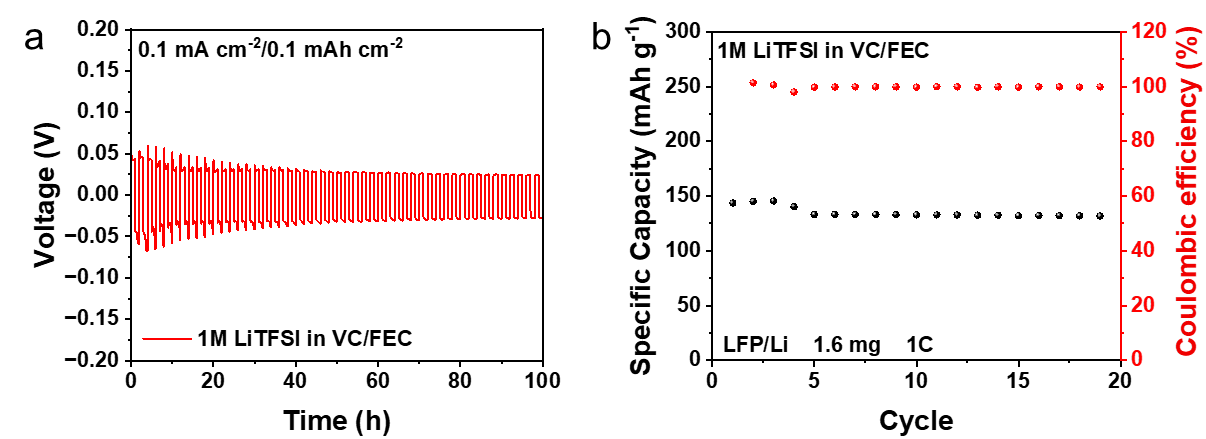
**

**Figure S22.** (a) Lithium stripping-plating profiles of Li/Li cell with precursor solution at a current density of 0.1 mA cm^−2^ with a cycling capacity of 0.1 mAh cm^−2^. (b) Room-temperature cyclic performance of LFP/Li cell with precursor solution at 1C.


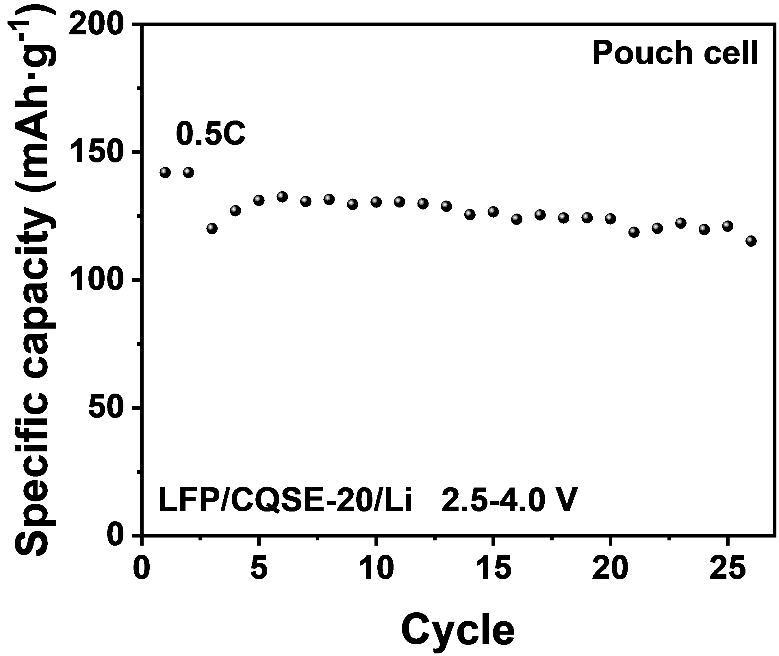


**Figure S23.** Cyclic performance of LFP/CQSE-20/Li pouch cell at room temperature.


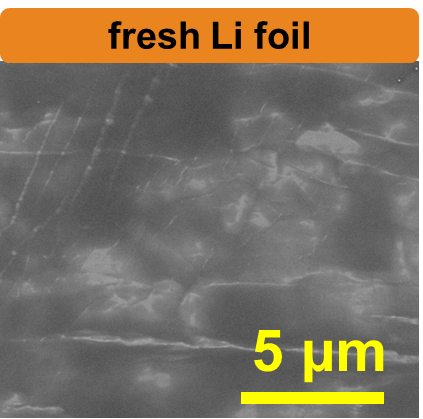


**Figure S24.** Top-view SEM images of fresh Li foil.


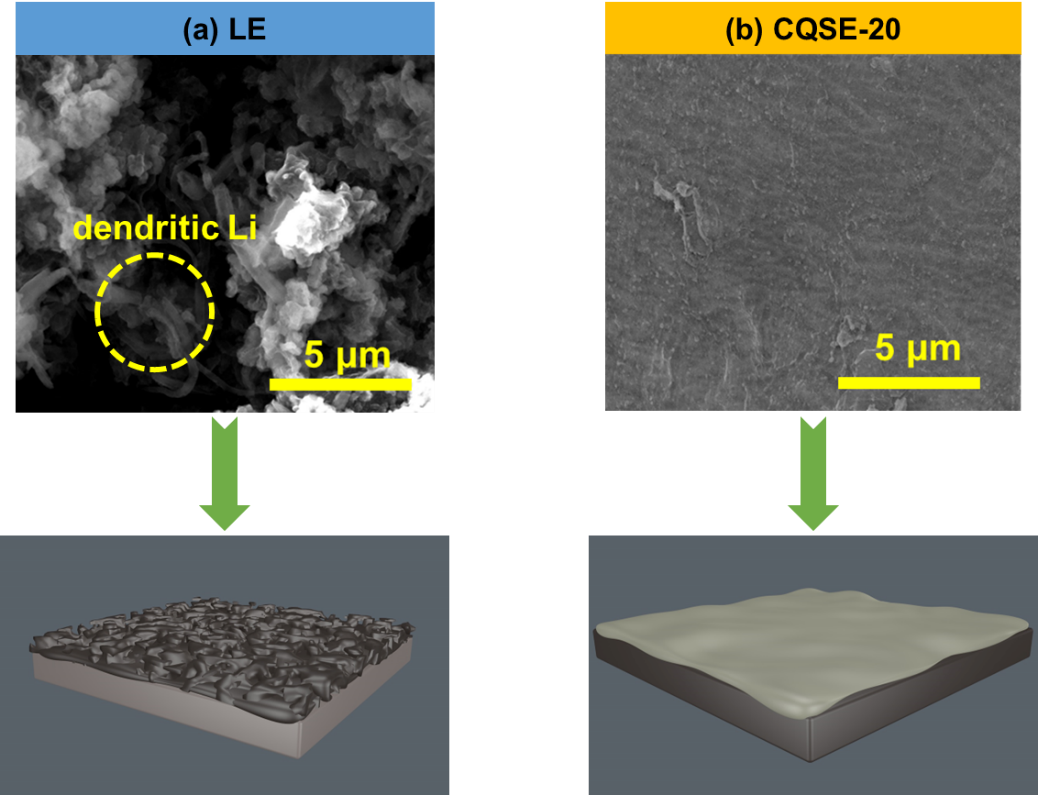


**Figure S25.** Top-view SEM images of Li foil and schematic illustration of lithium stripping-plating after 1000 cycles in LFP/Li cell at 1C using different electrolytes. (a) LE; (b) CQSE-20.


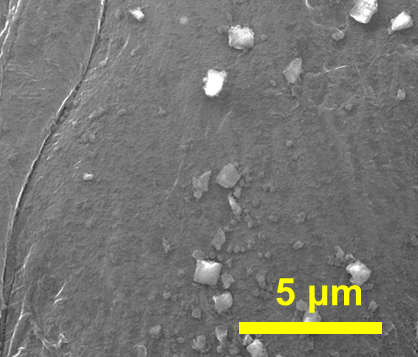


**Figure S26.** Top-view SEM images of Li metal anode after 1000 cycles from LFP/CQSE-20/Li cell at 4C.


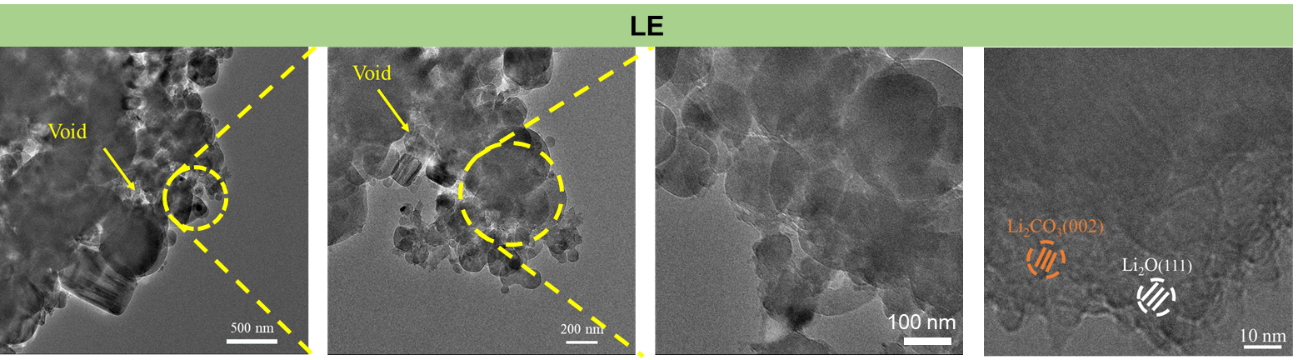


**Figure S27.** Cryo-TEM of Li deposition morphology and SEI structure using LE.


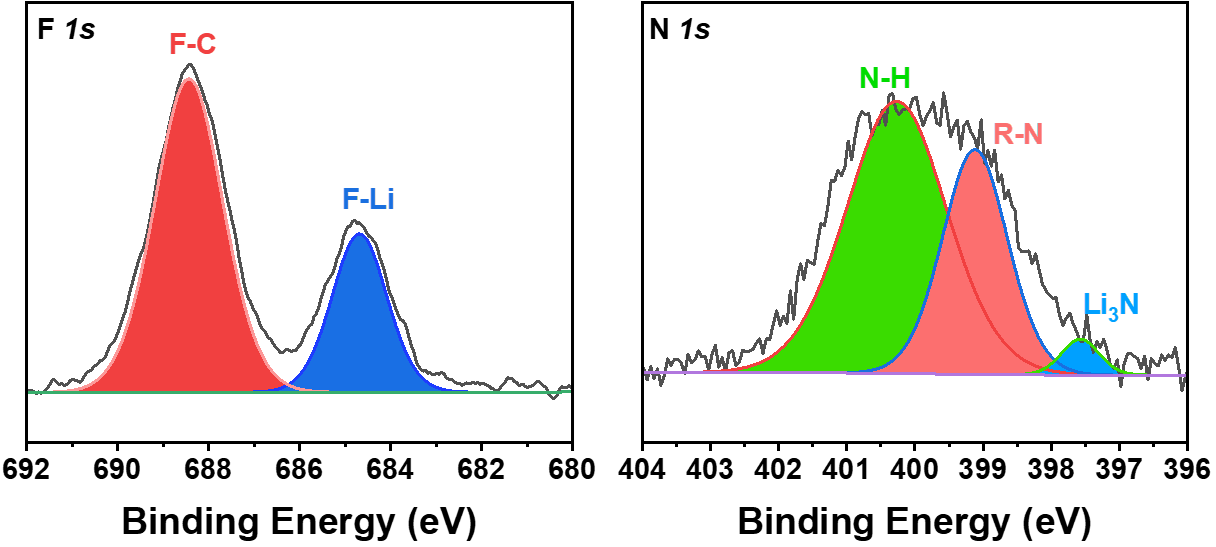


**Figure S28.** The XPS curves of the cycled Li metal anode after cycling 1000 h at 4C from the LFP/Li cell using CQSE-20.


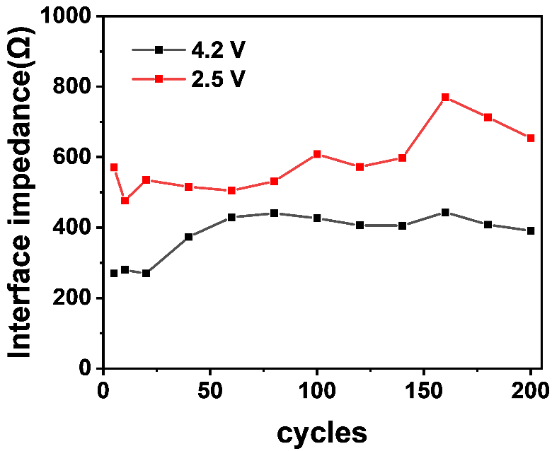


**Figure S29.** The interfacial impedance values of LFP/CQSE-20/Li cell measured at 4.2 V and 2.5 V with different cycles at 1C.


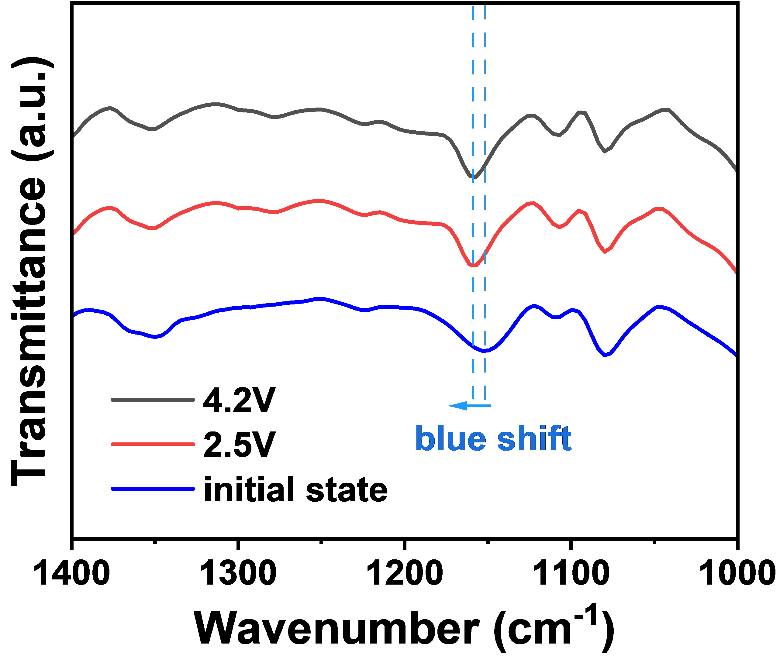


**Figure S30.** The FT-IR spectra of CQSE-20 measured at 4.2 V and 2.5 V after 200 cycles at 1C.


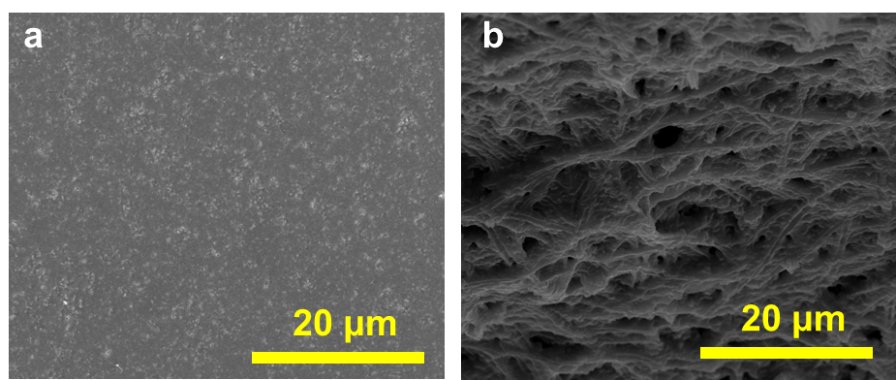


**Figure S31.** The SEM images of CQSE-20 after 1000 cycles at 1C from the LFP/Li cell: (a) top-view SEM image; (b) side-view SEM image.

**Table S1.** Comparison of the key properties of CQSE-20 electrolytes with previously reported solid electrolytes.

| **No.** | **Electrolytes** | **Burning test** | **σ (S·cm^-1^)** | **t_Li+_** | **Li/Li cells performance** | **LFP/Li batteries performance** | **Ref** |
| --- | --- | --- | --- | --- | --- | --- | --- |
| 1 | poly(ionic liquid)s-in-Salt/LATP | ─ | 0.17×10^-3^ (50 ℃) | ─ | 3500 h at 0.05 mA cm^-2^ (50 ℃) | 99.6% capacity retention after 100 cycles (0.1C, RT) | ^[2]^ |
| 2 | PVDF@LATP@PVDF | ─ | 6.84×10^-5^ (RT) | ─ | 3000 h at 0.1 mA cm^-2^ (RT) | 83.4% capacity retention after 300 cycles (0.2C, 60 ℃) | ^[3]^ |
| 3 | Janus LATP | ─ | 7.1×10 ^-4^ (60 ℃) | ─ | 3000 h at 0.1 mA cm^-2^ (60 ℃) | 89.9% capacity retention after 300 cycles (0.5C, 60 ℃) | ^[4]^ |
| 4 | BNRA-LATP/Li | ─ | 1.69×10^-4^ (RT) | ─ | 1800 h at 0.05 mA cm^-2^ (RT) | 92.0% capacity retention after 500 cycles (0.5C, RT) | ^[5]^ |
| 5 | LATP@B-TERB | ─ | 3.67×10^-4^ (RT) | ─ | 1800 h at 0.2 mA cm^-2^ (RT) | 78.6% capacity retention after 250 cycles (0.5C, RT) | ^[6]^ |
| 6 | LATP/F-HNBR | ─ | 6.3×10^-4^ (RT) | 0.79 | 1600 h at 0.1 mA cm^-2^ (RT) | 98.3% capacity retention after 500 cycles (0.5C, RT) | ^[7]^ |
| 7 | Ti-LiF LATP | ─ | ─ | ─ | 360 h at 0.1 mA cm^-2^ (RT) | 83.4% capacity retention after 60 cycles (0.2C, RT) | ^[8]^ |
| 8 | LATP-MoS_2_ @SP | ─ | 2.9×10^-4^ (RT) | ─ | 1200 h at 0.1 mA cm^-2^ (RT) | 86.2% capacity retention after 400 cycles (1C, 60 ℃) | ^[9]^ |
| 9 | GPE-PVDF@LATP | ─ | 1×10^-4^ (RT) | ─ | 1800 h at 0.1 mA cm^-2^ (RT) | 92% capacity retention after 400 cycles (0.5C, RT) | ^[10]^ |
| 10 | MLATP | ─ | 8.73×10^-4^ (RT) | ─ | 1000 h at 0.2 mA cm^-2^ (RT) | 80% capacity retention after 385 cycles (2C, 60 ℃) | ^[11]^ |
| **11** | **CQSE-20** | **UL-94**  **LOI (47%)** | **1.06×10^-3^ (RT)** | **0.63** | **750 h at 0.2 mA cm^-2^ (RT)** | **90% capacity retention after 1000 cycles (1C, RT);**  **86.9% capacity retention after 1000 cycles (4C, RT)** | **This work** |

**References**

[1] G. Wang, C. Chen, Y. Chen, X. Kang, C. Yang, F. Wang, Y. Liu, X. Xiong, Angew. Chem., Int. Ed. 2020, 59, 2055-2060.

[2] X. Song, C. Wang, J. Chen, S. Xin, D. Yuan, Y. Wang, K. Dong, L. Yang, G. Wang, H. J. A. F. M. Zhang, Adv. Funct. Mater. 2022, 32, 2108706.

[3] D. Wang, F. Zheng, Z. Song, H. Li, Y. Yu, X. J. I. Tao, E. C. Research, Ind. Eng. Chem. Res. 2022, 61, 14891-14897.

[4] F. Zheng, Z. Song, H. Li, Y. Z. Zheng, X. J. E. A. Tao, Electrochim. Acta 2022, 436, 141395.

[5] L. Zhu, Y. Wang, Y. Wu, W. Feng, Z. Liu, W. Tang, X. Wang, Y. J. A. F. M. Xia, Adv. Funct. Mater. 2022, 32, 2201136.

[6] T. Gu, L. Chen, Y. Huang, J. Ma, P. Shi, J. Biao, M. Liu, W. Lv, Y. J. E. He, E. Materials, Energy Environ. Mater. 2023, 6, e12531.

[7] Z. Geng, Y. Sun, Q. Zhang, S. P. Shen, L. Zhang, J. C. Zheng, Y. Luo, Y. Shi, Z. J. S. Chen, Small 2024, 2402041.

[8] X. Wang, X. Xu, W. Hou, Y. Chen, Y. Yang, Y. Wang, Z. Guo, Z. Song, Y. J. A. E. M. Liu, Adv. Energy Mater. 2024, 2402731.

[9] Q. Xia, S. Yuan, Q. Zhang, C. Huang, J. Liu, H. J. A. S. Jin, Adv. Sci. 2024, 2401453.

[10] D. Yang, Y. Yang, Y. Sun, T. J. A. F. M. Zhang, Adv. Funct. Mater. 2024, 2420202.

[11] C. Huang, S. Huang, A. Wang, Z. Liu, D. Pei, J. Hong, S. Hou, L. Vitos, H. Jin, Journal of Materials Chemistry A 2022, 10, 25500-25508.
